# Adaptation and Inhibition Control Pathological Synchronization in a Model of Focal Epileptic Seizure

**DOI:** 10.1523/ENEURO.0019-18.2018

**Published:** 2018-10-05

**Authors:** Anatoly Buchin, Cliff C. Kerr, Gilles Huberfeld, Richard Miles, Boris Gutkin

**Affiliations:** 1 University of Washington, Department of Physiology and Biophysics (United States, Seattle), 1959 NE Pacific St, 98195; 2 University of Sydney, School of Physics (Australia, Sydney), Physics Rd, NSW 2006; 3 Sorbonne Université-UPMC, Pitié-Salpêtrière Hô, Neurophysiology Department (France, Paris), 47-83 Boulevard de l’Hôpital, 75013; 4 Institut national de la santé et de la recherche médicale Unit 1129 “Infantile Epilepsies and Brain Plasticity”, Paris Descartes University, Sorbonne Paris Cité University group, (France, Paris), 149 rue de Sévres 75015; 5 Brain and Spine Institute, Cortex and Epilepsie Group (France, Paris), 47 Boulevard Hôpital, 75013; 6 Paris Sciences & Lettres Research University, Laboratoire des Neurosciences Cognitives, Group for Neural Theory (France, Paris), 29, rue d'Ulm, 75005 France; 7 National Research University Higher School of Economics, Center for Cognition and Decision Making (Russia, Moscow), 20 Myasnitskaya, 109316

**Keywords:** adaptation, AHP current, neural mass model, synaptic noise, temporal lobe epilepsy

## Abstract

Pharmacoresistant epilepsy is a common neurological disorder in which increased neuronal intrinsic excitability and synaptic excitation lead to pathologically synchronous behavior in the brain. In the majority of experimental and theoretical epilepsy models, epilepsy is associated with reduced inhibition in the pathological neural circuits, yet effects of intrinsic excitability are usually not explicitly analyzed. Here we present a novel neural mass model that includes intrinsic excitability in the form of spike-frequency adaptation in the excitatory population. We validated our model using local field potential (LFP) data recorded from human hippocampal/subicular slices. We found that synaptic conductances and slow adaptation in the excitatory population both play essential roles for generating seizures and pre-ictal oscillations. Using bifurcation analysis, we found that transitions towards seizure and back to the resting state take place via Andronov–Hopf bifurcations. These simulations therefore suggest that single neuron adaptation as well as synaptic inhibition are responsible for orchestrating seizure dynamics and transition towards the epileptic state.

## Visual Abstract

**Figure:**
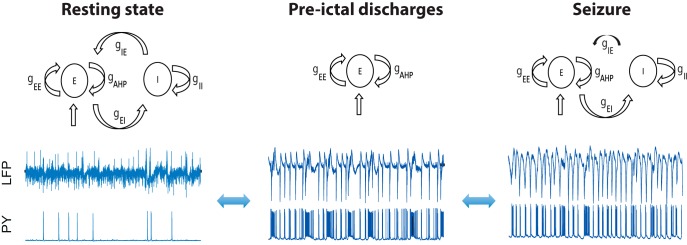

## Significance Statement

Epileptic seizures are commonly thought to arise from a pathology of inhibition in the brain circuits. Theoretical models aiming to explain epileptic oscillations usually describe the neural activity solely in terms of inhibition and excitation. Single neuron adaptation properties are usually assumed to have only a limited contribution to seizure dynamics. To explore this issue, we developed a novel neural mass model with adaption in the excitatory population. By including adaptation together with inhibition in this model, we were able to account for several experimentally observed properties of seizures, resting state dynamics, and pre-ictal oscillations, leading to improved understanding of epileptic seizures.

## Introduction

Epilepsy is the fourth most common neurologic disorder, and is responsible for a greater total global burden of disease than any neurologic conditions except for stroke and migraine (Beghi et al., 2005; Rothstein et al., 2005; Chin and Vora, 2014). Epileptic seizures are characterized by the increased excitability/excitation in the brain’s recurrently coupled neuronal networks (Lytton, 2008). Typically, experimental seizure models assume that seizures occur due to decreased inhibition (Karnup and Stelzer, 1999; Sivakumaran et al., 2015) or increased excitation in the neural networks (Ursino and la Cara, 2006; Hall and Kuhlmann, 2013).

There is also evidence that interneurons increase their firing at seizure initiation (Lillis et al., 2012) and are active during the time course of the epileptic activity (Ziburkus et al., 2006), suggesting that the activity of interneurons contributes importantly to aspects of seizure dynamics. The activity-dependent interplay between the pyramidal cells and interneurons could play an essential role for seizure generation mechanisms (Krishnan and Bazhenov, 2011; Naze et al., 2015; Buchin et al., 2016b). In neural mass models, neuron populations are often treated as rate units lacking intrinsic adaptation (Touboul et al., 2011). The dynamic behavior of the neural populations is determined by the balance between excitation and inhibition. Despite the simplicity of these models, they can be successfully used to reproduce resting and interictal states as well as ictal discharges by producing time series comparable with macroscopic measurements such as electroencephalogram signals and field potentials (Demont-Guignard et al., 2009).

However, not all types of epileptic seizures can be explained by looking only at the balance between excitation and inhibition (Traub et al., 2005); intrinsic excitability changes on the single-neuron level also play an important role (Krishnan and Bazhenov, 2011). Studies on human subiculum tissue showed that the complete blockade of type A GABAergic neurotransmission (and thus inactivation of the effects of inhibitory population) precludes seizure emergence while, if applied after seizure initiation, it abolishes rather than enhances the seizure activity. These manipulations usually bring back the neural network in the slice toward pre-ictal events, which have substantially different frequency content than seizure activity (Huberfeld et al., 2011), and which in this case fail to trigger ictal events. In human epileptic tissues, including peritumoral neocortex (Pallud et al., 2014), interictal discharges are generated spontaneously. These events are triggered by interneurons which depolarize pyramidal cells with impaired chloride regulation, leading to depolarizing effects of GABA. Once activated, pyramidal cells excite other cells via AMPA-mediated glutamatergic transmission. In these tissues, seizures can be produced by increasing local excitability using modified bathing media. The transition to seizures is characterized by the emergence of specific pre-ictal events initiated by pyramidal cells which synchronize local neurons by AMPA synapses. These pre-ictal events cluster before seizure initiation which requires functional AMPA, NMDA as well as GABA_A_ signals. The conventional neural mass models are unable to explain these pre-ictal oscillations because they require the excitatory population to generate periodic oscillations in the absence of inhibition. The second motivation for incorporating intrinsic excitability into neural mass models is that in epileptogenic areas, such as human subiculum, there is a substantial proportion of neurons with non-trivial intrinsic properties such as spike-frequency adaptation (Jensen et al., 1994; Huberfeld et al., 2007). To take these properties into account, neural mass models need to be enriched by the addition of components such as slow potassium currents (Pinsky and Rinzel, 1994).

In addition, seizures are typically accompanied by high potassium concentrations (Dietzel and Heinemann, 1986; Xiong and Stringer, 1999; Fröhlich and Bazhenov, 2006; Florence et al., 2009), which in turn activate calcium currents (Bazhenov et al., 2004; Fröhlich et al., 2008), which in turn affect spike-frequency adaptation and intrinsic bursting. These properties are likely to modulate the single neuron firing and thus further influence the neuronal dynamics. These findings motivate the development of neural mass models that can capture the intrinsic excitability in coupled neural populations.

In this work, we developed a novel neural mass model consisting of an inhibitory neural population and an adaptive excitatory neuronal population (Buchin and Chizhov, 2010b). We calibrated the parameters of the model to local field potential (LFP) data recorded in human subiculum slices during rest, seizure, and full disinhibition in pre-ictal condition. We then analyzed the model as calibrated to each of these three regimes. Our results emphasize the role of intrinsic excitability such as adaptation in the excitatory population, which help explain the transitions between rest, seizure, and full disinhibition states.

## Materials and Methods

### Epileptic tissue

Temporal lobe tissue blocks containing the hippocampus, subiculum, and part of the entorhinal cortex were obtained from 45 people of both sexes with pharmacoresistant medial temporal lobe epilepsies associated with hippocampal sclerosis (age, 18–52 years; seizures for 3–35 years) undergoing resection of the amygdala, the hippocampus, and the anterior parahippocampal gyrus. All of the individuals gave their written informed consent and the study was approved by the Comité Consultatif National d’Ethique.

### Tissue preparation

The post-surgical tissue was transported in a cold, oxygenated solution containing 248 mM D-sucrose, 26 mM NaHCO_3_, 1 mM KCl, 1 mM CaCl_2_, 10 mM MgCl_2_, and 10 mM D-glucose, equilibrated with 5% CO_2_ in 95% O_2_. Hippocampal-subicular-entorhinal cortical slices or isolated subicular slices (400-µm thickness, 3 × 12 mm length and width) were cut with a vibratome (HM650 V, Microm). They were maintained at 37°C and equilibrated with 5% CO_2_ in 95% O_2_ in an interface chamber perfused with a solution containing 124 mM NaCl, 26 mM NaHCO_3_, 4 mM KCl, 2 mM MgCl_2_, 2 mM CaCl_2_, and 10 mM D-glucose. Bicuculline or picrotoxin was used to block GABA_A_ receptors. Ictal-like activity was induced by increasing the external K^+^ concentration to 8 mM and reducing the Mg^2+^ concentration to 0.25 mM to increase the cellular excitability (similar to Huberfeld et al., 2011).

### Recordings

Up to four tungsten electrodes etched to a tip diameter of ∼5 µm were used for the extracellular recordings. The signals were amplified 1000-fold and filtered to pass frequencies of 0.1 Hz to 10 kHz (AM Systems, 1700). The extracellular signals were digitized at 10 kHz with a 12-bit, 16-channel A-D converter (Digidata 1200A, Molecular Devices) and monitored and saved to a PC with Axoscope (Molecular Devices).

### Data analysis

Records were analyzed using pCLAMP 10 software and scripts written in MATLAB 2016a. Power spectrum estimation was performed using fast Fourier transforms. The major frequencies of oscillations were computed via the multitaper method (Thomson, 1982).

### Simulations and analysis

Neural population model simulations were performed in XPPAUT 8.0 using the direct Euler method of integration, with a time step of 0.05 ms. Smaller time steps were tested and provided substantially similar results. In all simulations the initial conditions were systematically varied to check stability of numerical results. The data for the model was taken from one representative patient in the brain slice demonstrating resting state, seizure and pre-ictal oscillations.

### Software accessibility

The model code is available on GitHub (https://github.com/abuchin/EI-with-adaptation). Bifurcation analysis was performed in the XPP AUTO package (http://www.math.pitt.edu/~bard/xpp/xpp.html). All code is also available as Extended Data 1.

### Neural mass model

In the model we considered interacting excitatory and inhibitory neural populations coupled by AMPA and GABA_A_ synapses. All model parameters and variables are presented in Tables 1, 2. Each population was characterized by the average membrane potential of a population of leaky integrate-and-fire (LIF) neurons (similar to Chizhov and Graham, 2007; Touboul et al., 2011) with approximations for adaptive currents taken from Buchin and Chizhov (2010b):

$$C_{E}\frac{U_{E}}{dt}=I_{E}-I_{Na}^{LE}-I_{K}^{LE}-I_{Cl}^{LE}-g_{AHP}a\left(U_{E}-V_{AHP}\right)-g_{EE}e\left(U_{E}-V_{AMPA}\right)-g_{IE}i\left(U_{E}-V_{GABA}\right)$$

$$C_{I}\frac{U_{I}}{dt}=-I_{Na}^{LI}-I_{K}^{LI}-I_{Cl}^{LI}-g_{EI}e\left(U_{I}-V_{AMPA}\right)-g_{II}i\left(U_{I}-V_{GABA}\right)$$

where

$$I_{Na}^{LE/I}=g_{Na}^{LE/I}(U_{E/I}-V_{Na})$$

$$I_{K}^{LE/I}=g_{K}^{LE/I}(U_{E/I}-V_{K})$$

$$I_{Cl}^{LE/I}=g_{Cl}^{LE/I}(U_{E/I}-V_{Cl})$$

**Table 1.** Population model parameters

	Excitatory population	
Parameter	Value	Interpretation
$C_{E}$	1 mF/cm^2^	Membrane capacitance (Buchin and Chizhov, 2010b)
$g_{Na}^{LE}$	0.02 mS/cm^2^	Sodium leak conductance (Krishnan and Bazhenov, 2011)
$g_{K}^{LE}$	0.044 mS/cm^2^	Potassium leak conductance (Krishnan and Bazhenov, 2011)
$g_{Cl}^{LE}$	0.01 mS/cm^2^	Chloride leak conductance (Krishnan and Bazhenov, 2011)
$g_{AHP}$	1.6 mS/cm^2^	AHP-current conductance (Buchin and Chizhov, 2010b)
$g_{EE}$	1.5 mS/cm^2^	Excitatory-to-excitatory conductance
$g_{EI}$	1 mS/cm^2^	Excitatory-to-inhibitory conductance
$g_{IE}$	2; 0.5; 1 mS/cm^2^	Inhibitory-to-excitatory conductance
$g_{II}$	0.2 mS/cm^2^	Inhibitory-to-inhibitory conductance
$U_{reset}^{E}$	–65 mV	Reset membrane potential (Chizhov and Graham, 2007; Buchin and Chizhov, 2010b)
$V_{thr}^{E}$	–55 mV	Threshold membrane potential (Chizhov and Graham, 2007; Buchin and Chizhov, 2010b)
$a_{E}$	2.84 × 10^4^	Sigmoid fit parameter
$b_{E}$	0.19 mV^-1^	Sigmoid fit parameter
$c_{E}$	1.23 × 10^4^	Sigmoid fit parameter
$d_{E}$	–10 mV	Sigmoid fit parameter (threshold)
$\sigma_{E}$	3 μA/cm^2^	Input current variance
$\tau_{E}$	5.4 ms	AMPA current correlation time (Buchin et al., 2016a,b)
$\sigma_{VE}$	4 mV	Membrane potential dispersion
$V_{Na}^{E}$	50 mV	Sodium reversal potential (Krishnan and Bazhenov, 2011)
$V_{K}^{E}$	–75 mV	Potassium reversal potential (Krishnan and Bazhenov, 2011)
$V_{Cl}^{E}$	–93 mV	Chloride reversal potential (Krishnan and Bazhenov, 2011)
$V_{GABA}$	–75 mV	GABA reversal potential (Huberfeld et al., 2007)
$V_{AMPA}$	0 mV	AMPA reversal potential (Brunel and Wang, 2001)
$V_{AHP}$	–70 mV	AHP reversal potential (Brunel and Wang, 2001)
$\tau_{AHP1}$	1 ms	AHP rise time (Brunel and Wang, 2001)
$\tau_{AHP2}$	320 ms	AHP decay time (Brunel and Wang, 2001)
$\tau_{AMPA1}$	1 ms	AMPA rise time (Chizhov, 2002)
$\tau_{AMPA2}$	5.4 ms	AMPA decay time (Chizhov, 2002)
	**Inhibitory population**	
**Parameter**	**Value**	**Interpretation**
$C_{I}$	1 mS/cm^2^	Membrane capacitance (Buchin and Chizhov, 2010b)
$g_{Na}^{LI}$	0.02 mS/cm^2^	Sodium leak conductance (Krishnan and Bazhenov, 2011)
$g_{K}^{LI}$	0.04 mS/cm^2^	Potassium leak conductance (Krishnan and Bazhenov, 2011)
$g_{Cl}^{LI}$	0.03 mS/cm^2^	Chloride leak conductance (Krishnan and Bazhenov, 2011)
$g_{IE}$	2 mS/cm^2^	Inhibitory-excitatory synaptic conductance
$g_{II}$	0.2 mS/cm^2^	Excitatory-inhibitory synaptic conductance
$U_{reset}^{I}$	–65 mV	Reset membrane potential
$V_{thr}^{I}$	–55 mV	Threshold membrane potential
$a_{I}$	2.84 × 10^4^	Sigmoid fit parameter
$b_{I}$	0.19 mV^-1^	Sigmoid fit parameter
$c_{I}$	1.23 × 10^4^	Sigmoid fit parameter
$d_{I}$	–10 mV	Sigmoid fit parameter (threshold)
$\sigma_{VI}$	4 mV	Membrane potential dispersion
$V_{Na}^{I}$	50 mV	Sodium reversal potential (Krishnan and Bazhenov, 2011)
$V_{K}^{I}$	–75 mV	Potassium reversal potential (Krishnan and Bazhenov, 2011)
$V_{Cl}^{I}$	–82 mV	Chloride reversal potential (Krishnan and Bazhenov, 2011)
$\tau_{GABA1}$	8.3 ms	GABA-A decay time (Chizhov et al., 2002)
$\tau_{GABA2}$	0.2 ms	GABA-A rise time (Chizhov, 2002)

**Table 2.** Population model variables

**Variable**	**Interpretation**
$U_{E}$, mV	Average membrane potential of the excitatorypopulation
$U_{I}$, mV	Average membrane potential of the inhibitory population
e	Excitatory population synaptic gating variable
i	Inhibitory population synaptic gating variable
a	Excitatory population adaptation gating variable
$I_{E}(t)$, μA/cm^2^	Random excitatory input
$\nu_{E}\left(t\right)$, Hz	Firing rate of the excitatory population
$\nu_{I}\left(t\right)$, Hz	Firing rate of the inhibitory population

The firing rate of each population is computed based on the interspike interval distribution of the neural population (Gerstner and Kistler, 2002):

$$\nu_{E/I}\left(t\right)=A(U_{E/I})$$

where

$$A\left(U\right)={\left[\tau_{m}^{E/I}\int_{(U_{reset}^{E/I}-U_{E/I})/\sigma_{VE/I}}^{(V_{threshold}^{E/I}-U_{E/I})/\sigma_{VE/I}}e^{u^{2}}(1+erf(u))du\right]}^{-1}$$

and

$$\tau_{m}^{E/I}=\frac{C_{E/I}}{g_{Na}^{LE/I}+g_{K}^{LE/I}{+g}_{Cl}^{LE/I}}$$

In all simulations $\nu_{E/I}\left(t\right)$ has been approximated by the following sigmoid function:

$$\nu_{E/I}\left(t\right)=\frac{1}{\tau_{m}^{E/I}}\times \frac{a_{E/I}}{c_{E/I}+exp(-b_{E/I}(U_{E/I}+d_{E/I}))}$$

The population firing rate determines the adaptive (a), excitatory (e), and inhibitory (i) gating variables. Their dynamics are computed using the second-order approximation (Wendling et al., 2002; Chizhov, 2014):

$$\tau_{AHP1}\tau_{AHP2}\frac{d^{2}a}{{dt}^{2}}+{(\tau }_{AHP1}+\tau_{AHP2})\frac{da}{dt}+a=(1-a){\tau \nu }_{E}$$

$$\tau_{AMPA1}\tau_{AMPA2}\frac{d^{2}e}{{dt}^{2}}+{(\tau }_{AMPA1}+\tau_{AMPA2})\frac{de}{dt}+e=(1-e)\tau \nu_{E}$$

$$\tau_{GABA1}\tau_{GABA2}\frac{d^{2}i}{{dt}^{2}}+{(\tau }_{GABA1}+\tau_{GABA2})\frac{di}{dt}+i=(1-i)\tau \nu_{I}$$

To mimic the afferent excitatory input, the excitatory population also received stochastic excitatory input modeled as an Ornstein–Uhlenbeck process (Buchin and Chizhov, 2010a):

$$\tau_{E}\frac{{dI}_{E}}{dt}={-I}_{E}+\sigma_{E}\eta (t)$$

To mimic elevated extracellular potassium from epileptogenic slice experiments, in the population model, we increased potassium reversal potential in both populations $V_{K}^{E/I}$ from –90 to –75 mV, i.e., from $K_{o}=4$ mM to $K_{0}=8$ mM. This value of $V_{K}^{E/I}$ was computed based on Nernst equation, $V_{K}=\frac{RT}{F}ln(\frac{K_{o}}{K_{i}})$, where $\frac{RT}{F}=26.64$ mV and $K_{i}=138$ mM (Krishnan and Bazhenov, 2011).

All model parameter values and variable names are present in Table 1, 2. The initial parameter set was chosen manually to reproduce the pre-ictal like oscillations due to balance between $g_{EE}$ and $g_{AHP}$, seizure and resting state were fit such that $g_{EI}$ parameter variations would make a transition between seizure and resting state.

### LFP model

The LFP was calculated based on the activity of the excitatory population. We assumed that pyramidal cells activity dominates the extracellular field (Buzsáki et al., 2012). The dominant theory is that the LFP component is dominated by the single neuron dipole contribution (Buzsáki et al., 2012). Since the neural mass model averages over single neurons, the dipole moment cannot be directly modeled. Thus, to approximate the LFP being recorded near somas of the excitatory populations, we used the assumption that the average membrane potential of the excitatory population is proportional to the LFP, i.e., $LFP\propto U_{E}$ (Ursino and la Cara, 2006; Demont-Guignard et al., 2009; Wendling et al., 2012; Ratnadurai-Giridharan et al., 2014).

## Results

### Construction of the population model

**Figure 1.**
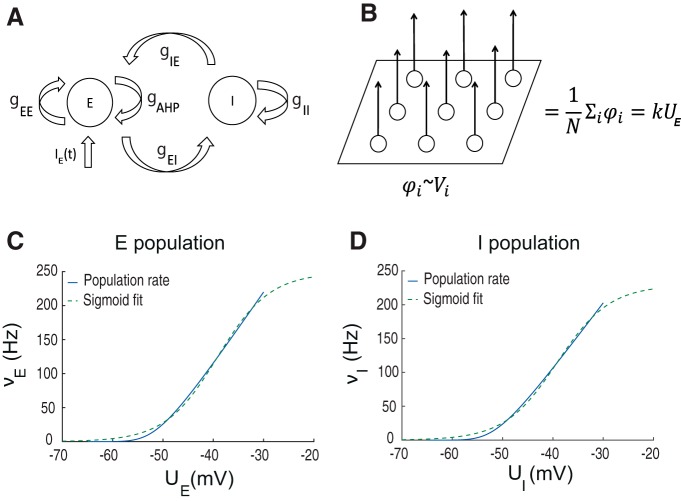
Structure of the population model. ***A***, Scheme of interacting neural populations. E, I: excitatory and inhibitory populations; $g_{EE}$, $g_{EI}$: excitatory to excitatory and excitatory to inhibitory maximal conductances; $g_{II}$, $g_{IE}$: inhibitory-to-inhibitory and inhibitory-to-excitatory maximal conductance; *g_AHP_*: adaptation conductance in the excitatory population; $I_{E}(t)$: synaptic noise input to the excitatory population; AHP, afterhyperpolarization current (Buchin and Chizhov, 2010b). ***B***, LFP model: $\varphi_{i}$: contribution of a single excitatory cell; *N*: the number of neurons; $U_{E}$: the average membrane potential in the excitatory population. ***C***, ***D***, Sigmoid approximation of potential-to-rate function (Johannesma, 1968) of the excitatory (***C***) and inhibitory population (***D***).

We developed а model of interacting excitatory and inhibitory population inspired by Wilson–Cowan approach (Wilson and Cowan, 1972), which consists of excitatory and inhibitory populations coupled by synaptic connections (Fig. 1*A*). The firing rate in each population depends on the average membrane potential $U_{E/I}$, which is governed by the subthreshold dynamics of LIF neuron population similar to (Gerstner and Kistler, 2002; Chizhov, 2014; as explained in Materials and Methods). Firing rates of the excitatory and inhibitory populations are determined using the values of $U_{E/I}$ put through function $A(U_{E/I})$ (Johannesma, 1968; Gerstner and Kistler, 2002). To make the model numerically stable and amenable to bifurcation analysis we used a sigmoid function to estimate the population firing rate provided by the $A(U_{E/I})$ approximation. To justify the choice of sigmoid parameters, we used least-squares to match it with the analytical solution (Johannesma, 1968; Fig. 1*C*,*D*). The sigmoid approximation allows one to efficiently take into account zero and linear parts of the potential-to-rate transfer functions $v_{E/I}(t)$, and provides saturation due to the single neuron refractory period (Renart et al., 2004). The sigmoid functions of excitatory and inhibitory populations are shown in Figure 1*C*,*D*. The difference between the excitatory and inhibitory populations was taken into account by adjusting passive conductances for sodium, potassium, and chloride leak currents estimated in Krishnan and Bazhenov, (2011) based on dynamic ion concentration model.

The subthreshold $U_{E/I}$ dynamics determine the synaptic $g_{EE}e(\upsilon_{E})$, $g_{EI}e(\upsilon_{E/I})$, $g_{II}i(\upsilon_{I})$, $g_{IE}i(\upsilon_{I})$, and intrinsic $g_{AHP}e(\upsilon_{E/I})$ conductances (Fig. 1*A*), computed according to the population firing-rates $\upsilon_{E/I}$. Similar to spiking neural network models (Bazhenov et al., 2004; Ratnadurai-Giridharan et al., 2014), adaption in our population model reduces neural firing in the excitatory population after periods of activity. Excitatory population receives external random synaptic input to model excitation from the rest of the brain similar to (Jansen and Rit, 1995; Touboul et al., 2011). To mimic the experimental epileptogenic conditions of human subiculum slice experiments, the potassium reversal potential was elevated from –95 to –75 mV both in the excitatory and inhibitory populations to provide excitatory drive to reproduce the experimental conditions. Elevation of extracellular potassium also leads the increase of intracellular chloride reducing the efficiency of inhibition due to elevated GABA_A_ reversal potential (Huberfeld et al., 2007; Buchin et al., 2016b). To generate the model output comparable with experimental data, we computed the LFP generated by the excitatory population (Buzsáki et al., 2012). This approximation assumes that all pyramidal cells in the excitatory population contribute equally to the recorded LFP signal (Fig. 1*B*). Thus, the total LFP near somas depends on the average value of the membrane potential in the excitatory population with a certain dimensionality constant, i.e., $LFP\propto kU_{E/I}$.

### Reproduction of epileptic oscillations

**Figure 2.**
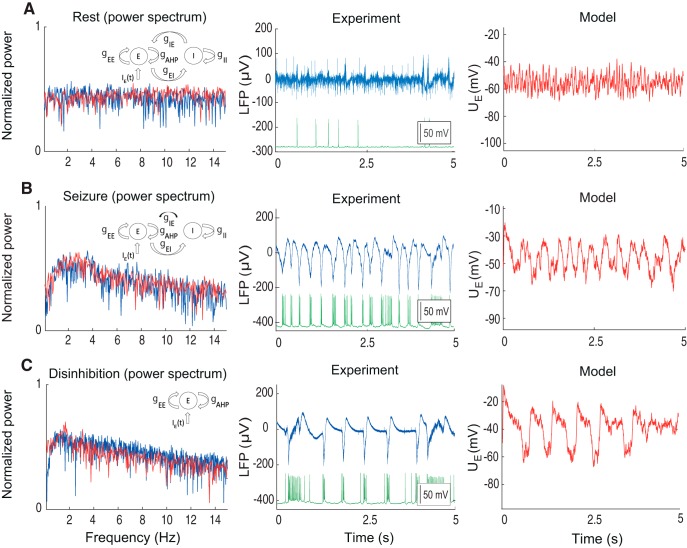
Neural mass model in various excitatory regimes. ***A***, Activity of a neural population in the resting state. ***B***, Seizure state. ***C***, Disinhibited state. LFP is present together with intracellular recording from the pyramidal cell. Each plot contains the model scheme, power spectrum, and time traces provided by the excitatory population $U_{E}$ as well as experimental LFP. Red traces correspond to the model, blue traces to the experiment, and green traces to the intracellular recordings from the pyramidal cells. Model parameters for (***A***): $g_{EE}$ = 1.5 mS/cm^2^; $g_{EI}$ = 1 mS/cm^2^; $g_{IE}$ = 2 mS/cm^2^; $g_{II}$ = 0.2 mS/cm^2^; $g_{AHP}$ = 1.6 mS/cm^2^; (***B***): $g_{EE}$ = 1.5 mS/cm^2^; $g_{EI}$ = 1 mS/cm^2^; $g_{IE}$ = 0.5 mS/cm^2^; $g_{II}$ = 0.2 mS/cm^2^; $g_{AHP}$ = 1.6 mS/cm^2^; (***C***): $g_{EE}$ = 1.5 mS/cm^2^; $g_{EI}$ = 1 mS/cm^2^; $g_{IE}$ = 0 mS/cm^2^; $g_{II}$ = 0.2 mS/cm^2^; $g_{AHP}$ = 1.6 mS/cm^2^.

When the excitatory and inhibitory synaptic currents were dynamically balanced, the activity stayed in the low-firing regime, as indicated by LFP power spectrum (Fig. 2). The recorded pyramidal cell during this period demonstrated sparse firing activity, partially time-locked with the discharges on the LFP. We call this activity in the model the balanced or resting state (Fig. 2*A*). In this regime, the model does not generate epileptic oscillations. To evaluate the model performance in this resting state, we compared the synthetic LFP with the experimental LFP recorded between seizures (Fig. 2*A*). Similar to the experimental data, we found that in the resting state, the model generates broadband oscillations, with the highest power in the 1- to 15-Hz frequency band. In this regime, the average membrane potential of the excitatory population $U_{E/I}$ stays in the range from –60 to –50 mV.

We found that the model was not capable of generating interictal discharges using this parameter set. It has been recently suggested that interneurons play the key role in generating interictal activity (Cohen et al., 2002; Huberfeld et al., 2011). In the presence of GABA_A_ blockade these events were completely blocked, indicating that they depend on combination of GABAergic and glutamatergic signaling. In the recent population model (Chizhov et al., 2017), it was proposed that interictal discharges could be initiated by the inhibitory population, thus explaining interneuron firing before pyramidal cell firing (Huberfeld et al., 2011). In our model we have not explored this scenario, i.e., when the inhibitory population is also receiving the background synaptic input. These mechanisms would likely play an important role for seizure initiation; however, incorporating all mechanisms at once would make the model impossible to study analytically. Therefore, we have not considered interictal discharges before seizure, while aiming to specifically describe other types of oscillations.

To reproduce the seizure state in the model, we reduced the synaptic inhibition of the excitatory population by decreasing the synaptic conductance parameter $g_{IE}$ (Fig. 2*B*, black arrow). All other parameters of the model remained the same. In this case the model moved into an oscillatory regime in which the power spectrum of the oscillations changed dramatically to include strong oscillations in the 1- to 4-Hz frequency band, which is typical for ictal discharges (Huberfeld et al., 2011).

**Figure 3.**
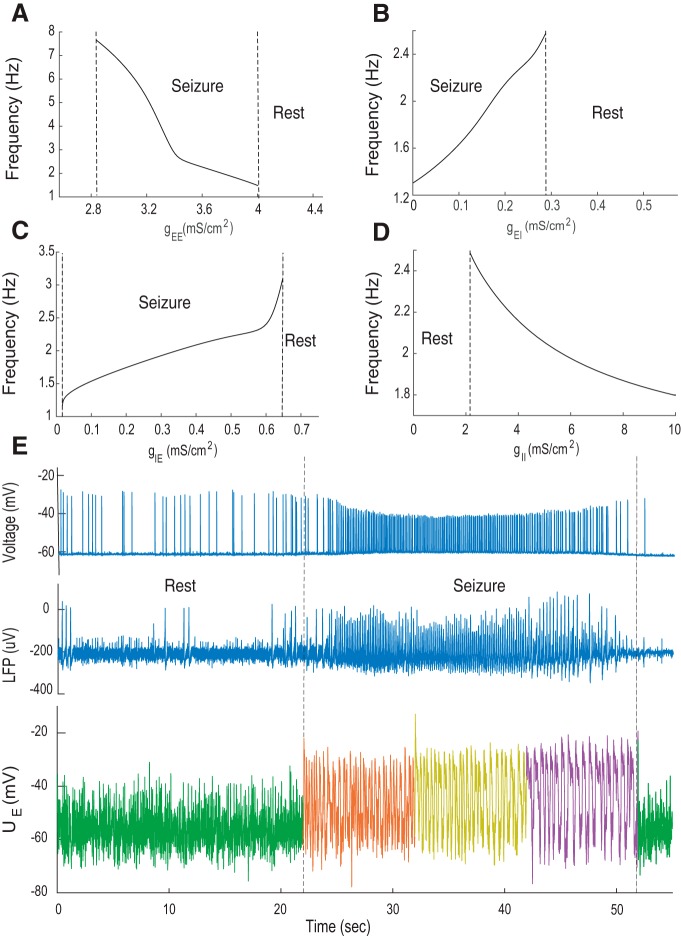
Oscillatory frequencies of the population model. ***A*–*D***, Oscillatory frequencies of the population model in the absence of the synaptic noise ($I_{E}(t)$ = 0) as a function of the synaptic conductance, $g_{EE},g_{EI},g_{IE}{,g}_{II}$. ***E***, Simultaneous intracellular recording from single pyramidal cell, LFP, and population model during transition from the resting state toward seizure. States marked by dotted lines. The green trace corresponds to the model’s resting state (Fig. 2*A*, $g_{IE}=2\;mS/{cm}^{2}$), red corresponds to early seizure (Fig. 2*B*, $g_{IE}=0.5\;mS/{cm}^{2}$), yellow corresponds to late seizure ($g_{IE}=0.25\;mS/{cm}^{2}$), and purple corresponds to the disinhibition state (Fig. 2*C*, $g_{IE}=0\;mS/{cm}^{2}$).

We compared the model power spectrum with the measured LFP recorded during the initial phase of the ictal discharge with the hypersynchronous activity onset. During this activity regime the recorded pyramidal cells generated strong bursts of spikes temporally locked to the LFP (Fig. 2*B*). The population model displayed discharges with the same frequency band as in the LFP, indicating large amount of synchrony in the excitatory population (Buzsáki et al., 2012). Note that we considered only the initial phase of the seizure (the whole ictal event is shown in Fig. 3*E*).

To further test the validity of our model, we explored its dynamics with inhibitory activity completely blocked (Fig. 2*C*). In these simulations the initial conditions were set to the resting state and parameter values of the model were set to the seizure state, but with the conductance $g_{IE}$ (from the inhibitory to the excitatory population) set to zero to mimic the experimental conditions. In this case the GABAergic effects of the inhibitory population in the slice has been fully blocked by bicuculine after seizures have been previously established (Huberfeld et al., 2011). In response to this change, the activity in the slice became highly synchronized and reduced to regular pre-ictal discharges. During these oscillations the pyramidal cells generated large bursts of activity, temporally coupled with the LFP (Fig. 2*C*). In the model, similarly to the experimental preparation, the blockade of the GABAergic signaling mimicked by the abolition of the inhibitory population led to the development of a slow oscillatory rhythm with a peak frequency around 1 Hz. These events have been previously reported as pre-ictal discharges (Huberfeld et al., 2011). This rhythm has much slower frequency than seizures, and is usually within the 1- to 4-Hz frequency range (Huberfeld et al., 2011; Buchin et al., 2016b). In addition, these events recur regularly for long periods with very limited modulation.

We call this regime of activity pre-ictal discharges because similar activity takes place before transition toward an ictal state (Huberfeld et al., 2011). In this regime, the dynamics of the excitatory population are determined only by the balance between self-excitation, $g_{EE}e(\upsilon_{E/I})$, afterhyperpolarization current (AHP; Chizhov and Graham, 2008; Buchin and Chizhov, 2010b), $g_{AHP}e(\upsilon_{E/I})$, and the afferent synaptic current $I_{E}(t)$. Hence, these pre-ictal oscillations in the model are driven by the synaptic noise and adaptation. The excitatory input to the excitatory population $I_{E}(t)$ drives the upswings of $U_{E}$ due to recurrent excitatory synapses, with activity then being terminated by AHP currents. These transitions take place randomly due to stochastic nature of the synaptic input.

For quantitative comparisons between the model and experiment we used the linear fit to the power spectrum over frequencies and peak estimation (Table 3). We found that there is substantial intersection between linear fits applied to the power spectrums in resting, seizure, and pre-ictal states (Fig. 2). We found that there is a substantial overlap between these frequencies, providing validation for the model. Note that we compared the overall spectral characteristics between the model and experiment by variation of only one parameter, $g_{IE}$ to reproduce transitions between the pre-ictal, resting and seizure states. If more parameters are varied at the same time, it would be possible to get a better match between the model and experiment.

**Table 3.** Power spectrum analysis

	Model, peak amplitude, Hz	Experiment, peak amplitude, Hz	Model, spectrum linear fit, 1/Hz	Experiment, spectrum linear fit, 1/Hz
Rest	-	-	-0.005—-0.002	-0.005—-0.002
Seizure	3.01—3.52	2.95—3.75	-0.005—-0.002	-0.003—-0.002
Pre-ictal state	1.33—1.43	1.21—1.79	-0.007—-0.003	-0.01—-0.008

Overall oscillations in our population model are controlled by the balance between synaptic currents, adaptation and external synaptic input. When synaptic and intrinsic conductances are balanced, the population demonstrates resting state activity, characterized by a flat power spectrum. When there is an imbalance between excitation and inhibition, populations start developing oscillatory rhythms associated with ictal discharges with a frequency of 3–4 Hz. However, complete loss of inhibition leads to the development of another population rhythm, pre-ictal discharges with 1-Hz frequency, controlled by adaptation and recurrent excitation. Thus, the dynamic state of a neural population depends on the interplay between the intrinsic and synaptic excitability within populations as well as external synaptic input.

### Analysis of the population model

To delineate the mechanisms giving rise to the different oscillatory modes in the model, we used continuation techniques and bifurcation analysis. Since it is impossible to use the standard techniques to identify bifurcations in the presence of noise, we analyzed the model in the absence of an external input $I_{E}(t)$. This allowed us to compute the model behavior in the stationary regime and characterize bifurcations happening during transitions between different oscillatory regimes. The initial parameters were chosen to correspond to the resting state. The parameter variations were calculated around this point in the parameter space for $g_{EE}$, $g_{EI}$, $g_{IE}$, and $g_{II}$ bifurcation diagrams, with other parameters held fixed. Analysis of $g_{AHP}$ and $V_{GABA}$ variations was implemented for another parameter set, where $g_{IE}=0.5$ μS/cm^2^ and $g_{IE}=1$ μS/cm^2^; other parameters remained the same.

The frequency of seizure oscillations depends on the strength of the synaptic currents in the population model. There is a nonlinear relationship between seizure major frequency and the recurrent excitatory conductance $g_{EE}$ (Fig. 3*A*). When the $g_{EE}$ is increased up to 2.8 mS/cm^2^, the model responds with an oscillatory frequency near 7.5 Hz. When self-excitation is further increased up to 4 mS/cm^2^, seizure-related oscillations disappear since the system moves to the high activity state due to sigmoidal saturation of the transfer function (Fig. 1*C*,*D*). The amount of stimulation of the inhibitory population also influences the oscillatory frequency. When $g_{EI}$ is in the range of 0 to 0.29 mS/cm^2^ (Fig. 3*B*), the population model generates seizure activity with frequencies of 1.2–2.5 Hz. Note that seizure oscillations are possible even when $g_{EI}=0$ mS/cm^2^.

Inhibitory synaptic connections also affect the oscillatory frequency of seizure activity. When $g_{IE}$ is as low as ∼0.6 mS/cm^2^ (Fig. 3*C*), the seizure activity starts around 3 Hz; it decreases to ∼1 Hz when $g_{EI}$ is close to zero (when $g_{IE}=0$ mS/cm^2^, there is no seizure activity in the model). The amount of recurrent inhibition also determines the seizure oscillatory frequency (Fig. 3*D*). Seizure activity can be initiated by sufficient self-inhibition, i.e., when $g_{II}$ is near 2 mS/cm^2^, seizures of 2.5 Hz are observed. When $g_{II}$ increases, the seizure frequency decreases; for example, at 10 mS/cm^2^, seizure activity is ∼1.8 Hz.

In the previous sections, the population model was calibrated to data for short periods of seizure activity, where the frequency was not substantially changing (Fig. 2*B*). Yet, one can see that in the experiment, seizure activity is not stationary and its frequency changes over time. The time course of a typical seizure is shown in Figure 3*E*. Before the seizure starts there is a resting state, characterized by occasional interictal (Cohen et al., 2002) and pre-ictal discharges (Huberfeld et al., 2011). When seizure starts at 22 s, it is characterized by fast oscillations of the extracellular field in the range of 5–6 Hz in the initial phase. During the time course of seizure activity, it gradually decreases to 1-Hz frequency, and from 52 s, it gradually stops.

We aimed to reproduce this aspect of seizure activity using the population model (Fig. 3*E*). First, the model was initialized in the resting state (Fig. 2*A*), green trace. Second, we reduced the amount of inhibitory-to-excitatory coupling (to $g_{IE}=0.5\;mS/{cm}^{2}$) to reproduce the seizure state, red trace. Third, we gradually reduced the coupling parameter (to $g_{IE}=0.25\;mS/{cm}^{2}$) to reduce the oscillation frequency, yellow trace. Fourth, to model the slow oscillations in the end of seizure, we set the coupling parameter to zero ($g_{IE}=0\;mS/{cm}^{2}$), violet trace. Finally, we restored it to the original value to bring the model back to rest ($g_{IE}=2\;mS/{cm}^{2}$), green trace. This example illustrates how the transition toward seizure in the population model can be achieved by varying only one parameter, the inhibitory-to-excitatory conductance $g_{IE}$.

**Figure 4.**
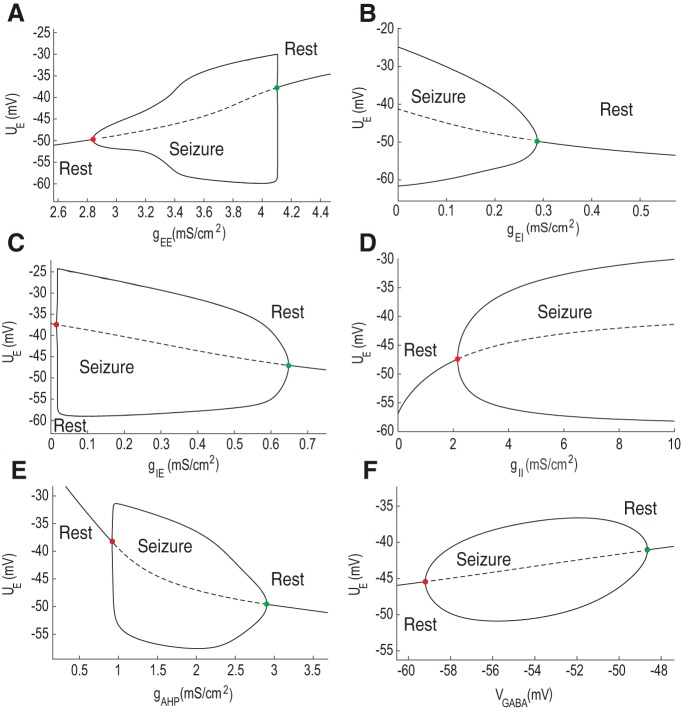
Analysis of the population model. ***A–D***, Bifurcation diagrams for the variations of the maximal synaptic conductances, including recurrent excitation $g_{EE}$, excitation from excitatory to inhibitory population $g_{EI}$, inhibition from inhibitory to excitatory population $g_{IE}$, and the recurrent inhibition in the inhibitory population $g_{II}$, respectively. ***E***, ***F***, Bifurcation diagrams for adaptation in the excitatory population $g_{AHP}$ and GABA reversal potential $V_{GABA}$ from the inhibitory-to-excitatory current, $g_{IE}i(U_{E}-V_{GABA})$. Diagrams ***A–D*** were calculated for $g_{IE}$ = 2 mS/cm^2^; ***E***, *g_IE_* = 0.5 mS/cm^2^; and ***F***, *g_IE_* 1 mS/cm^2^. The value of $U_{E}$ characterizes the average membrane potential in the resting state and maximal/minimal values of $U_{E}$ during the oscillations. Red and green dots correspond to the supercritical and subcritical Andronov–Hopf bifurcations. Solid and dotted lines depict the stable and unstable solutions.

To study the amplitude of pathologic oscillations, we performed a bifurcation analysis and tracked changes of the average membrane potential in the excitatory population, $U_{E}$ (Fig. 4), the self-excitation conductance $g_{EE}$ (Fig. 4*A*). We found that increasing $g_{EE}$ leads to the development of ictal oscillations when its value increases beyond ∼2.8 mS/cm^2^. During the gradual increase of $g_{EE}$, the constant steady state loses stability via the supercritical Hopf bifurcation (Izhikevich, 2007), red dot. After passing this point the neural populations start developing seizure oscillations. This activity regime is stable for large $g_{EE}$ variations, implying that seizure dynamics are possible for a large range of recurrent excitation. When $g_{EE}$ becomes higher than a critical value (>4.1 mS/cm^2^) and the system loses stability via the subcritical Hopf bifurcation, green dot. It corresponds to the high activity state with no oscillations. This happens due to the sigmoid approximation of the population rate (Johannesma, 1968), when $\nu_{E/I}$ reaches the saturation level (Fig. 1*C*,*D*).

Second, we considered the excitatory to inhibitory conductance $g_{EI}$ (Fig. 4*B*). In this case, seizure activity is blocked when $g_{EI}$ is larger than 0.3 mS/cm^2^. If $g_{EI}$ is smaller than 0.3 mS/cm^2^, it leads to seizure activity via a subcritical Hopf bifurcation, green dot. Similar to the $g_{EE}$ bifurcation diagram, seizure dynamics are possible for a large range of $g_{EI}$. These results show that a decrease in the excitatory conductance from excitatory to inhibitory populations is sufficient to provoke seizure activity. Note that even if $g_{EI}=0$ mS/cm^2^, the excitatory population still receives the input from the inhibitory one because potassium reversal potential is elevated. These changes in potassium reversal potential drive both excitatory and inhibitory population even if synaptic drive is not present. For example, when $g_{EI}$ = 0 mS/cm^2^, the increased potassium reversal potential still drives the inhibitory population, providing the inhibitory input to the excitatory population. It happens because it decreases the leak current thus depolarizing the membrane potential of excitatory and inhibitory neurons. Therefore, seizure oscillations are still present because inhibition is still present. Seizure frequency in this case is near 1.25 Hz (Fig. 3*B*) and $U_{E}$ oscillates between –61 and –25 mV.

Third, we considered inhibitory-to-excitatory conductance $g_{IE}$ (Fig. 4*C*). When $g_{IE}=0$, the model shows resting state activity. This corresponds to the condition when the inhibitory population does not have any influence on the excitatory one. Experimentally this scenario is achieved when inhibitory neurotransmission is completely blocked. Therefore, in the complete absence of inhibition, seizure activity could not be generated. In turn, pre-ictal oscillations are not possible without the contribution of the external synaptic noise $I_{E}(t)$ when $g_{IE}=0$ mS/cm^2^. When there is stochastic synaptic input, it occasionally brings the system to the oscillatory regime associated with seizures (Fig. 2*C*). Then oscillations are promoted due to recurrent excitation and terminated via AHP adaptation mechanism. Thus, without synaptic stimulatin of the inhibitory population, the model is incapable of seizure generation. In turn, pre-ictal oscillations do not require inhibition, but strongly depend on the recurrent excitatory-to-excitatory connections $g_{EE}$, adaptation $g_{AHP}$, and the external synaptic input $I_{E}(t)$. When inhibitory to excitatory conductance $g_{IE}$ becomes strong enough, around 0.65 mS/cm^2^, seizure oscillations become truncated and the system moves back to the resting state via subcritical Andronov–Hopf bifurcation, green dot.

Fourth, we evaluated the role of recurrent inhibitory conductance $g_{II}$ for seizure dynamics (Fig. 4*D*). When there is substantial amount of self-inhibition in the inhibitory population, it leads to an increase of excitation in the whole system because of synaptic coupling. If $g_{II}$ is above 2.1 mS/cm^2^, it leads to the development of seizure oscillations via a supercritical Hopf bifurcation, red dot. Seizure activity in this case persists for the large variations in $g_{II}$ variations, from 2.2 to >10 mS/cm^2^.

We then analyzed the effect of adaptation in the excitatory population. We found the regime in the parameter space of the model for which $g_{AHP}$ becomes the critical parameter for seizure oscillations. To find this regime we slightly modified the parameter set, where $g_{IE}$ = 0.5 mS/cm^2^ instead of 2 mS/cm^2^. In this case, $g_{AHP}$ could substantially affect seizure oscillations. When $g_{AHP}$ is in the range of 1–3 mS/cm^2^, there is a large region in the parameter space that produces seizure oscillations. If $g_{AHP}$ is larger than 3 mS/cm^2^, the seizure dynamics becomes truncated due to the inhibitory effect of adaptation via subcritical Andronov–Hopf bifurcation, green dot. Yet when adaptation is not strong enough, $g_{AHP}$ is lower, the model demonstrates seizure oscillations. If $g_{AHP}$ is lower than 1 mS/cm^2^, seizure oscillations become impossible and the model moves to the high activity state without oscillations via supercritical Andronov–Hopf bifurcation. Additionally, we found that in the complete absence of adaptation, seizure oscillations are still possible in the model (results not shown), but pre-ictal oscillations could not be generated because of the absence of negative feedback provided by adaptation. To be able to reproduce seizure oscillations together with pre-ictal oscillations induced by GABA_A_ blockade, adaptation in the excitatory population is required.

We further studied the critical role of $V_{GABA}$ for seizure generation. It has been recently found that changes in $V_{GABA}$ are associated with the rhythm generation in the hippocampus (Cohen et al., 2002; Huberfeld et al., 2007). The analysis was performed for slightly modified parameter set, where $g_{IE}$=1 mS/cm^2^, such that $V_{GABA}$ becomes the bifurcation parameter. The other parameters remained the same. We have changed the initial parameter set to find the region of the parameter space where $V_{GABA}$ could play the crucial role for oscillations. We found that when $V_{GABA}$ is higher than –59 mV, it leads to ictal oscillations (Fig. 4*F*). If $V_{GABA}$ drops below –48 mV, the oscillations stop. These transitions take place due to supercritical and subscritical Andronov–Hopf bifurcations. Thus, there is substantial range of $V_{GABA}$ where its increase leads to the development of seizures, which might take place due to chloride accumulation before an ictal discharge (Huberfeld et al., 2007; Lillis et al., 2012).

In summary, using bifurcation analysis, we characterized the parameter regions of the model where seizure oscillations could take place. We found that transitions from seizure to rest and from rest to seizure take place via supercritical and subcritical Andronov–Hopf bifurcations. In all studied cases we found that resting and oscillatory solutions exist for large parameter variations, implying the stability of found solutions (Prinz et al., 2004; Marder and Taylor, 2011). We showed that variations of synaptic $g_{EE}$, $g_{EI}$, $g_{IE}$, $g_{II}$, and intrinsic conductances $g_{AHP}$ could bring the system toward seizure and move it back to the resting state. It implies that combination of recurrent synaptic currents and spike-frequency adaptation in the excitatory population accounts for the transitions between seizure and resting states.

## Discussion

The objective of this study was to investigate the role of intrinsic excitability and inhibition as mechanisms of seizure dynamics. We constructed a novel neural mass model, consisting of interacting excitatory and inhibitory neural populations driven by external synaptic input. By comparing the model with the LFP data from human hippocampal/subicular slices, we found that it could accurately represent resting states, ictal discharges, and pre-ictal oscillations after the blockade of inhibition (Huberfeld et al., 2011). Analysis of the model showed that synaptic and intrinsic conductances play the most crucial role for transitions between resting and seizure activity. By analyzing the parameter space of the model, we found the oscillatory regimes specific for the resting state and seizure dynamics, and found that transitions between these regimes take place via subcritical and supercritical Andronov–Hopf bifurcations.

Starting with the pioneering work of Wilson and Cowan (Wilson and Cowan, 1972), neural mass models have traditionally aimed to reduce the complexity of neural dynamics toward interactions between excitation and inhibition. This approach has been validated in multiple studies for describing the large-scale brain activity patterns (Jirsa et al., 2010). Additionally, it has been shown that intrinsic properties of single neurons such as spike-frequency adaptation (Fröhlich et al., 2008) substantially change spiking patterns and thus neural dynamics (Kager et al., 2000; Bazhenov et al., 2004; Buchin et al., 2016b). So far these types of interactions have not been explicitly taken into account in neural mass models.

In this work, we developed a novel mass model by adding AHP currents (Buchin and Chizhov, 2010a) to the excitatory population. This allowed to efficiently take into account not only seizure and resting state dynamics (Wendling et al., 2012) but also pre-ictal oscillations. In our model seizure activity takes place due to imbalance between self-excitation, adaptation and inhibition. We found that reducing the amount of inhibition to the excitatory population provokes seizure activity. Nonetheless, inhibition plays an important role in orchestrating seizures as well (Fig. 2*B*). We found that the complete lack of inhibition leads to the development of slow oscillations with significantly different frequency content than seizures (Fig. 2*C*). Thus, we propose that inhibition, together with single neuron intrinsic properties provided by adaptation, plays an important role controlling the seizure dynamics.

We have investigated multiple mechanisms responsible for generation of seizure activity. In the proposed model, seizure oscillations could be generated by increased recurrent excitation $g_{EE}$, decreased excitation of the inhibitory population $g_{IE}$, decreased inhibition of the excitatory population $g_{IE}$, increased recurrent inhibition in the inhibitory population $g_{II}$. Changes in the intrinsic excitability of the excitatory population such as decrease of intrinsic adaptation $g_{AHP}$ and increase of the GABA_A_ reversal potential $V_{GABA}$ could also lead to seizure oscillations. We speculate that various physiological parameters combinations could lead to seizure activity, as found by Jirsa et al. (2014). The combination of multiple factors such as increased chloride concentration in the pyramidal cells and GABA_A_ reversal (Huberfeld et al., 2011; Lillis et al., 2012; Buchin et al., 2016a), together with an increase in extracellular potassium concentrations (Bazhenov et al., 2004; Krishnan and Chizhov, 2011) and decreased activity of interneurons (Ziburkus et al., 2006), all contribute to seizure initiation. Combination of these factors and their relative contribution should be evaluated via additional experiments and modeling.

Adaptation on the single neuron level could be achieved by calcium-dependent potassium currents (Jung et al., 2001; Bazhenov et al., 2004). In our model, AHP is the key mechanism for termination of population bursts during seizure oscillations (Fig. 2*B*) and pre-ictal discharges (Fig. 2*C*). The alternative potential mechanism of termination of these bursts are GABA_B_ currents provided by the inhibitory population (de la Prida et al., 2006). We predict that in the complete absence of the inhibitory neurotransmission including GABA_A_ and GABA_B_ synapses, the purely excitatory network in the epileptogenic slice of human subiculum would be capable of generating self-sustained pre-ictal oscillations due to negative feedback provided by AHP (Ratnadurai-Giridharan et al., 2014) and other intrinsic adaptation currents. Therefore the downregulation of excitatory neuronal adaptation currents such as AHP and/or functionally similar muscarinic-sensitive potassium currents (Stiefel et al., 2008) could lead to seizure initiation. According to the model the pharmacological strategy aiming to increase the amount of adaptation in the excitatory population would lead to the decreased susceptibility toward seizures.

GABA_B_ inhibition could also participate for the termination of population bursts. As shown by (de la Prida et al., 2006), the joint blockade of GABA_A_ receptors by PTX and GABA_B_ receptors by CGP led to generation of all-or-none population bursts in CA3 mouse hippocampal slices. In our experiments we did not test for the possibility that GABA_B_ could participate for the pre-ictal discharge termination. Additional experiments are needed to divide the contributions of GABA_B_ and AHP for the burst termination.

Note that oscillations in the slice switched from ictal discharges to pre-ictal ones after full GABA_A_ blockade. This transition was possible only if seizures were already established in the slice (Huberfeld et al., 2011). It implies that there are excitability and synaptic plasticity changes in the slice associated with seizures before the pre-ictal discharges could be established using complete GABA_A_ blockade. When GABA_A_ blockers were applied before first seizure being generated, the pre-ictal and ictal oscillations were not established (Huberfeld et al., 2011).

Our model has several limitations compared to existing approaches (Molaee-Ardekani et al., 2010; Wendling et al., 2012; Jirsa et al., 2014). First, it is unable to describe the pre-ictal discharges taking place before seizure. The work of Buchin et al. (2016b) proposes a network explanation of pre-ictal discharges that take place before seizure transition (Huberfeld et al., 2011). To describe this activity, it was necessary to take into account the heterogeneity in the excitatory population caused by depolarizing GABA, while in the current model we did not take it into account. Therefore, pre-ictal discharges in our model could be generated only in the absence of inhibitory population. Second, particular features such as high frequency oscillations (Engel et al., 2009) relevant for seizure initiation (Quilichini et al., 2012) are not captured in our model. We speculate that this property could be taken into account by incorporating fast somatic and slow dendritic inhibition (Wendling et al., 2012). Third, our model is also unable to describe the interictal discharges, which have been explained in the other population models (Wendling et al., 2012; Chizhov et al., 2017). It has been found that interictal discharges in human subiculum require initial interneuron activation. Since in our model we impose the background synaptic input onto the excitatory population, the pyramidal cells are always activated before interneurons. It has been recently proposed in Chizhov et al. (2017) that the interneuron population should receive background synaptic input, which would allow the reproduction of interictal discharges in neural mass models.

Pre-ictal discharges are generated before seizure initiation and in the absence of inhibition when seizures have been established in the slice (Huberfeld et al., 2011). These oscillations are still generated in the absence of inhibitory population (Fig. 2*C*). Using the model, we show that in this case the background synaptic input to the excitatory population $I_{E}(t)$ is necessary to generate the periodic pre-ictal oscillations. When $I_{E}(t)$ is absent, there are no pre-ictal discharges in the model (Fig. 3*C*). We speculate that before seizure initiation the interneurons are becoming non-functional because of depolarization block (Ziburkus et al., 2006) and GABA_A_ reversal (Lillis et al., 2012), thus allowing the pre-ictal discharges to be generated before seizure initiation (Huberfeld et al., 2011). The proposed model could explain the presence of pre-ictal discharges only in the complete absence of inhibition (Fig. 2*C*). The possibility of pre-ictal discharge generation before seizure due to non-functional inhibition could be investigated in the future studies.

During seizures or ictal discharges, the frequency content of spiking activity might substantially change (Fig. 3*E*). This can be explained using the current model as due to the gradual increase of recurrent excitation $g_{EE}$ (Fig. 3*A*) or the increase of recurrent inhibition $g_{II}$. Note that the frequency content of seizure oscillations in the end of it might be similar to the pre-ictal discharges (Figs. 2*C*, 3*E*). However, pre-ictal oscillations are possible in the model only in the absence of inhibition (Fig. 2*C*), as in the experimental data when the GABA_A_ synaptic activity is completely blocked.

The primary advantage of our model compared to more abstract ones such as Jirsa et al. (2014) is that it provides more firm biophysical explanations linking single neuron properties to population dynamics (Johannesma, 1968; Gerstner and Kistler, 2002; Chizhov and Graham, 2007). Our approach could be extended to take into account the shunting effect of inhibition by adjusting the firing rate transfer function (Chizhov et al., 2014). To describe the additional mechanisms of seizure transition, the present model could include slow activity-dependent parameter changes similar to (Cressman et al., 2009; Ullah et al., 2009; Proix et al., 2014; Chizhov et al., 2018). There are multiple biophysical mechanisms that could play the role of slow variable bringing the network toward seizure (Naze et al., 2015), including dynamic ion concentration of extracellular potassium (Bazhenov et al., 2004), intracellular chloride (Jedlicka et al., 2011; Buchin et al., 2016b), and intracellular sodium (Krishnan and Bazhenov, 2011; Karus et al., 2015), in pyramidal cells. The population model could be further modified to incorporate these slow mechanisms to describe seizure initiation.

A common problem with neural mass models in general is their limited ability to generate the experimentally measurable signals (Lytton, 2008). In this work, we used the average voltage of the excitatory neural population as the approximation of the LFP signal near the neurons’ somas (Ratnadurai-Giridharan et al., 2014). Given the distant dependence of the LFP signal, the current model should be considered only as a first approximation (Buzsáki et al., 2012). More detailed approaches describing populations of two-compartmental neurons (Chizhov, 2014; Chizhov et al., 2015) could also provide better approximation for the LFP.

Epilepsy is a complex phenomenon involving the dynamic interactions between multiple components of the nervous system (Lytton, 2008). In this work, we have investigated the particular role of inhibition and adaptation and their implications for seizure dynamics. Reconciling modeling results with experimental data, we have shown that seizure activity cannot be generated in the complete absence of the inhibitory population and adaption in the excitatory population. Further development of theoretical and experimental approaches in epilepsy research may lead to a better understanding of its mechanisms and the development of new therapeutic targets.

10.1523/ENEURO.0019-18.2018.ed1 Extended Data 1 The code is available as Extended Data. Download Extended Data, ZIP file

## Synthesis

Reviewing Editor: William Stacey, University of Michigan Decisions are customarily a result of the Reviewing Editor and the peer reviewers coming together and discussing their recommendations until a consensus is reached. When revisions are invited, a fact-based synthesis statement explaining their decision and outlining what is needed to prepare a revision will be listed below. The following reviewer(s) agreed to reveal their identity: Jonathan Touboul, Timothée Proix.

Two reviewers and I have discussed your paper and we find that it is of interest, particularly for the care in which you compared computational results with human data. The primary concern is that there are several statements that are not well supported by your data and analysis. Listed below under “Major”, these are statements in which you make strong conclusions about seizure mechanisms that sometimes ignore other possibilities. These concerns will require either additional work or else a reformulation in what is claimed/proven by the paper. In particular, Items 2 and 3 will require some additional work. Items 1, 4, and 6 deal with preictal discharges and can be addressed by reformulation or more analysis depending if you want to maintain the claim (i.e. as currently written, it would require more evidence to support the claims, but the claims could be reformulated and not provide additional evidence). Item 5 can be simply reformulated.

Major:

1. The authors call “preictal discharges” the limit cycle regime of the LFP when inhibition is removed from the model. As predicted by dynamical system theory, a recovery variable is necessary to create this limit cycle. The authors claim that this recovery variable is driven by spike-frequency adaptation mechanisms. While Huberfeld et al. (2011) have indeed shown that preictal discharges do not depend on GABAA signaling , GABAB signals could be responsible for this recovery variable dynamics. This statement, as noted by the authors, is supported by experiments (de la Prida et al., 2006), and theory (Wendling et al., 2002). Is there any further experimental or theoretical considerations that could further support the authors claim, that the dynamics of the recovery variable are driven by spike-frequency adaptation mechanisms, and not by GABAB signaling?

2. Along the same lines, the authors state that “adaptation in the excitatory population is an essential component of seizure generation mechanism, without it seizures could not be generated”. However they only show that no oscillations are generated for a specific set of conductance parameters. Additionally, these parameters were different from the rest of the analysis conducted in Fig. 4. A more convincing proof would be to set the AHP-current conductance to zero and systematically vary all the other conductance parameters (in the 4D space) and show that no pathological oscillations can appear. While I understand that such exploration may be computationally intractable, at least a 1D exploration of the different parameters for gAHP = 0 would help better supporting the author's claim.

3. To trigger ictal discharges, synaptic inhibition was reduced. However, it seems (please clarify this point in the Results section) that the ictal discharges were triggered in the experiments by increasing the K+ concentration, and reducing the Mg2+ concentration, which would correspond in the model to increased cellular excitability. The bifurcation diagram in Fig4A confirms that oscillatory activity can also simply be triggered by increasing self-excitation. Would this be another possible path to enter the seizure? More generally, while multiple paths can lead to oscillations through change of the studied parameters, are all these paths physiologically valid? Can the authors formulate constraints to identify scenarios that are physiologically relevant?

4. The authors choose to call preictal discharges the activity they observe when synaptic inhibition was blocked. One could also interpret this activity as being late spike-and-wave discharges happening towards the end of the seizure. Indeed, the frequency of the discharges tends to decrease towards the end of the seizure, down to 1Hz sometimes. The absence of inhibition would then correspond to the inhibitory neurons being in a depolarization block state. Furthermore, the model only displays such activity if the network is already initialized in the ictal state. Is there any further evidence that this activity resembles more preictal discharges than ictal spike-and-wave discharges?

5. The abstract mentions that synaptic noise was essential for seizure generation, but it is not clear how that is demonstrated in the text.

6. The model appears to have been validated by qualitative comparison of power spectra and time series between simulated and real data. Is there a way to quantify it? My suggestion is to use some basic features (band power, peak frequency, skewness, kurtosis, etc) of your current data that clearly show the distinction between A, B, and C in Figure 2, but show similarity between the model and the real data.

Minor:

1. The introduction should focus more on the novelty of the current paper with regards to excitability, and focus less on the excitation/inhibition balance in past studies (see R1 point #1)

2. Be accurate in descriptions of past models (R1 point #2).

3. Computer code should have a simple README to explain what each file does.

4. Reviewer 2 has a list of minor items below that should be corrected.

Below are the original reviews

Reviewer 1:

This is a very good paper on models of cortical assemblies. It is particularly well written and tests a clear and interesting hypothesis, namely the implication of adaptation and excitability in epileptic seizures. Also, a notable interest of this paper is the constant care for interpreting in a relevant manner computational findings, and the comparison with human data.

The code was uploaded in a zip file. It is clear and well designed. I tested the ode file with the parameter set of the seizure and it worked properly. Moreover, the raw data and the Matlab code for analysis of the results was provided. Comment: The Matlab code is not as straightforward to use as the ode code, as for instance spect_full.m requires defining UE and dt (which I did by loading the appropriate raw data files) and uncommenting a plot command. Overall, this is a very good data sharing.

Review of eNeuro Manuscrit eN-NWR-0019-18 :

Adaptation and inhibition control pathological synchronization in a model of focal epileptic seizure

This paper extends existing models of neural activity and combines the newly defined model with data on human epileptic tissues to address the role of various electro-physiological parameters in the emergence and termination of epileptic seizures. In particular, the paper discusses explicitly the role of intrinsic excitability and adaptation in the generation of epileptiform activity, which was never addressed at this level of detail in the literature.

Overall, the paper is very well written, the authors have made a notable effort of clarity. Another remarkable quality of this work is the constant connection made between the model's parameters and their electrophysiological counterparts. While the contribution uses up-to-date techniques of analysis of non-linear ODEs, this connection with biology makes the paper perfectly accessible to biologists, as well as to the computational neuroscience community.

Another remarkable aspect of the contribution is the comparison between model and human data, and the accurate qualitative fit between model and experiments. This gives the model important substance and realism.

Eventually, a very good discussion is provided about the limitations of the model used, as well as way to improve that model for future work. This very precise discussion is of quality and accurate.

This paper was overall very nice to read. There was, as indicated, a clear effort to explain the problem, the state of the art, the features of the model and the connection to electrophysiological quantities in the most intelligible manner. The results are new as far as I can tell, and provide a new light on a clear and important question raised in the introduction, namely the role of intrinsic excitability in epileptiform activity.

I believe this paper is a nice addition to the literature and will be appreciate in the computational neuroscience community, and therefore recommend publication.

I provide below a list of suggested revisions to improve the writing, essentially in the introduction, as the rest of the paper is remarkably clear and accurate.

Minor:

1. the introduction focuses on the balance between excitation and inhibition as the key point in previous studies, but I disagree with that statement. Imbalances were taken into account in almost all models cited, and those give rise to seizure-like activity. I would advise the authors to rather focus on the novelty of their approach, namely the introduction and analysis of excitability, which seems to the Referee to be the key element in their paper and the central novelty compared to the literature.

2. Previous models are sometimes described not very accurately. For instance, Touboul's et al model is not based on spiking units, but on a rate model introduced by Jansen & Rit. If the Referee is correct, both papers looked at changes in excitation/inhibition balance and how those changed activity patterns. I would suggest the authors to be more precise in their description.

Reviewer 2:

This new model is a valuable addition to the field. In particular, an interesting aspect of this work is the possibility of the model parameters to be directly derived from experimental data, and therefore to easily relate results of the model simulation/analysis to testable experiments. Using this model, the authors argue that spike-frequency adaptation is a critical component for the generation of preictal spikes. However, I do not think this claim is strongly supported by the evidence provided in this work, and that more analysis is required.

Code was downloaded and tested for the direct simulations of the model (Fig. 2). I did not reproduced the bifurcation diagrams (Fig. 3 and 4).

A README file and some cleaning of the code would be appreciated.

In this manuscript, the authors propose a new neural mass model to explore the role of spike-frequency adaptation into triggering pathological seizure activity. They investigate how different conductance parameters of this model can generate oscillatory activity using bifurcation diagrams constructed by continuation methods.

This new model is a valuable addition to the field. In particular, an interesting aspect of this work is the possibility of the model parameters to be directly derived from experimental data, and therefore to easily relate results of the model simulation/analysis to testable experiments. Using this model, the authors argue that spike-frequency adaptation is a critical component for the generation of preictal spikes. However, I do not think this claim is strongly supported by the evidence provided in this work, and that more analysis is required.

Major concerns

1. The authors call “preictal discharges” the limit cycle regime of the LFP when inhibition is removed from the model. As predicted by dynamical system theory, a recovery variable is necessary to create this limit cycle. The authors claim that this recovery variable is driven by spike-frequency adaptation mechanisms. While Huberfeld et al. (2011) have indeed shown that preictal discharges do not depend on GABAA signaling , GABAB signals could be responsible for this recovery variable dynamics. This statement, as noted by the authors, is supported by experiments (de la Prida et al., 2006), and theory (Wendling et al., 2002). Is there any further experimental or theoretical considerations that could further support the authors claim, that the dynamics of the recovery variable are driven by spike-frequency adaptation mechanisms, and not by GABAB signaling?

2. Along the same lines, the authors state that “adaptation in the excitatory population is an essential component of seizure generation mechanism, without it seizures could not be generated”. However they only show that no oscillations are generated for a specific set of conductance parameters. Additionally, these parameters were different from the rest of the analysis conducted in Fig. 4. A more convincing proof would be to set the AHP-current conductance to zero and systematically vary all the other conductance parameters (in the 4D space) and show that no pathological oscillations can appear. While I understand that such exploration may be computationally intractable, at least a 1D exploration of the different parameters for gAHP = 0 would help better supporting the author's claim.

3. To trigger ictal discharges, synaptic inhibition was reduced. However, it seems (please clarify this point in the Results section) that the ictal discharges were triggered in the experiments by increasing the K+ concentration, and reducing the Mg2+ concentration, which would correspond in the model to increased cellular excitability. The bifurcation diagram in Fig4A confirms that oscillatory activity can also simply be triggered by increasing self-excitation. Would this be another possible path to enter the seizure? More generally, while multiple paths can lead to oscillations through change of the studied parameters, are all these paths physiologically valid? Can the authors formulate constraints to identify scenarios that are physiologically relevant?

4. The authors choose to call preictal discharges the activity they observe when synaptic inhibition was blocked. One could also interpret this activity as being late spike-and-wave discharges happening towards the end of the seizure. Indeed, the frequency of the discharges tends to decrease towards the end of the seizure, down to 1Hz sometimes. The absence of inhibition would then correspond to the inhibitory neurons being in a depolarization block state. Furthermore, the model only display such activity if the network is already initialized in the ictal state. Is there any further evidence that this activity resembles more preictal discharges than ictal spike-and-wave discharges?

5. In the abstract, the authors mention that the synaptic noise was essential for seizure generation. I may have missed it, but it does not seem to me that this manuscript demonstrates this result.

6. Unless I missed something, the validation of the model with the recorded data is only based on the qualitative comparison of power spectra and time series. Could the authors find quantitative support to validate the agreement between model with the data?

Minor issues

- The Method section mentions that data from 45 patients were examined. How so? I only see example traces for a couple of patients. Were any parameters fitted to the data of these 45 patients?

- Please describe how the power spectrum was computed. The code shows that fft was used. Estimators that address bias/variance issues should be used instead, e.g. multitaper methods (Mitra & Pesaran 2011) .

- How were the parameters for the conductances chosen in the first place?

-When were the NBQX and d,I-AP5 used? Please describe in the legend of Fig. 2 the conditions used in each experiment, as well as the values of gIE used in the simulations.

- What does it mean for the model to be initialized in the seizure state? Are the initial conditions set differently?

- For the analysis of gAHP and VGABA, how were the values of gIE chosen? Why wasn't gIE set to 0, as suggested by the experiments?

- Page 10: what are vE, vE/I and vI?

- Page 17: gIE should be gEI.

- Page 17: please clarify “the increased potassium reversal potential still drives the inhibitory population?” What does drive mean in dynamical terms here?

- Page 18: I do not see how Fig. 4C demonstrates the assertion “it occasionally brings the system to the oscillatory regime associated with seizure”.

- Page 20: Fig. 4E should be Fig. 4F

- Fig. 2: the seizures appear to have a hypersynchronous onset (as opposed to a low-voltage fast activity onset). Does that mean this model is restricted to this type of seizure? If so, please mention it.

- Legend of Fig. 2: describe the blue and red traces. The left plot title should be “Power spectrum”, not the name of the state. Use different colors for LFP and intracellular recordings and describe them in the legend.

- Fig. 4A: shouldn't the “Rest” label be for low values of gEE?

- Fig. 4: does the stable fixed point on the other side of the seizure correspond to saturation of the model?

- Table 1: references for Ureset and Vthreshold?

- Table 1: typo: Interpretation for gIE: conuctance

- Table 2: I(t) should be IE(t)

- INa, IK, ICl and the firing rate are not described in Table 2.

- Bartolomei et al. (2014) and Proix et al. (2014) refer to the same paper. I would also suggest citing work Ullah et al. (2009) and Cressman et al. (2009).

## Author Response

We would like to thank the reviewers for their hard work and patience reviewing our manuscript. Suggested comments helped us to improve the clarity of the manuscript text and figures as well as to distill the key scientific ideas. To improve the quality of our work and address the reviewer's concerns, we have performed the following corrections:

Major

1. The authors call “preictal discharges” the limit cycle regime of the LFP when inhibition is removed from the model. As predicted by dynamical system theory, a recovery variable is necessary to create this limit cycle. The authors claim that this recovery variable is driven by spike-frequency adaptation mechanisms. While Huberfeld et al. (2011) have indeed shown that preictal discharges do not depend on GABAA signalling, GABAB signals could be responsible for this recovery variable dynamics. This statement, as noted by the authors, is supported by experiments (de la Prida et al., 2006), and theory (Wendling et al., 2002). Is there any further experimental or theoretical considerations that could further support the authors claim, that the dynamics of the recovery variable are driven by spike-frequency adaptation mechanisms, and not by GABAB signalling?

-&gt; We wish to specify that preictal discharges are not the activity generated by GABAA receptors inhibition but rather the epileptic activity induced by increase in neuronal excitability remaining after GABAA receptors inhibition.

In the work of de la Prida 2006 it has been shown that the blockade of GABAB receptors leads to generation of all-or-none hippocampal population bursts in hippocampal CA3 slices.

Initially the population bursts were induced by application of PTX to block the GABAA receptors. After population bursts have been established, GABAB blocker CGP has been added to the slices. We interpret this data that after the blockade of GABAA and GABAB inhibition, the population bursts could be generated due to intrinsic properties of single pyramidal neurons in CA3 area.

In our current experimental data, we have not tested the GABAB receptor blocker to test this particular hypothesis. To correct for the statement mentioned in the paper we did the following changes of the manuscript text in the Discussion section.

Corrections:

GABAB inhibition could also participate for the termination of population bursts. As shown by (de la Prida et al. 2006), the joint blockade of GABAA receptors by PTX and GABAB receptors by CGP led to generation of all-or-none population bursts in CA3 mouse hippocampal slices. In our experiments we did not test for the possibility that GABAB could participate for the pre-ictal discharge termination. Further experiments are needed to divide the contributions of GABAB and AHP for the burst termination.

2. Along the same lines, the authors state that “adaptation in the excitatory population is an essential component of seizure generation mechanism, without it seizures could not be generated”. However, they only show that no oscillations are generated for a specific set of conductance parameters. Additionally, these parameters were different from the rest of the analysis conducted in Fig. 4. A more convincing proof would be to set the AHP-current conductance to zero and systematically vary all the other conductance parameters (in the 4D space) and show that no pathological oscillations can appear. While I understand that such exploration may be computationally intractable, at least a 1D exploration of the different parameters for gAHP = 0 would help better supporting the author's claim.

-&gt; We have performed the parameter exploration for gAHP = 0. The results show that seizure-like oscillations are possible in the model even in the absence of AHP mechanism. The analysis showed that seizure-like oscillations could be generated in the absence of adaptation, i.e. when g_AHP=0 uS/cm^2. To make this point less controversial we made the following additions to the manuscript.

Corrections:

Additionally, we found that in the complete absence of adaptation, seizure oscillations are still possible in the model (results not shown), but pre-ictal oscillations could not be generated. To be able to reproduce seizure oscillations together with pre-ictal oscillations induced by GABAA blockade, adaptation in the excitatory population is required.

3. To trigger ictal discharges, synaptic inhibition was reduced. However, it seems (please clarify this point in the Results section) that the ictal discharges were triggered in the experiments by increasing the K+ concentration, and reducing the Mg2+ concentration, which would correspond in the model to increased cellular excitability. The bifurcation diagram in Fig4A confirms that oscillatory activity can also simply be triggered by increasing self-excitation. Would this be another possible path to enter the seizure? More generally, while multiple paths can lead to oscillations through change of the studied parameters, are all these paths physiologically valid? Can the authors formulate constraints to identify scenarios that are physiologically relevant?

The clarify the mechanisms of seizure initiation in the slice preparations we made the following corrections in the Materials and Methods section

Corrections:

Ictal-like activity was induced by increasing the external K+ concentration to 8 mM and reducing the Mg2+ concentration to 0.25 mM to increase the cellular excitability (similar to Huberfeld et al., 2011).

To make the point about multiple paths towards transition to seizure we made the following corrections:

Corrections:

We have investigated multiple mechanisms responsible for generation of seizure activity. In the proposed model, seizure oscillations could be generated by increased recurrent excitation g_EE, decreased excitation of the inhibitory population g_IE, decreased inhibition of the excitatory population g_IE, increased recurrent inhibition in the inhibitory population g_II. Changes in the intrinsic excitability of the excitatory population such as decrease of intrinsic adaptation g_AHP and increase of the GABAA reversal potential V_GABA could also lead to seizure oscillations. We speculate that various physiological parameters combinations could lead to seizure activity, as found by Jirsa et al. (2014). The combination of multiple factors such as increased chloride concentration in the pyramidal cells and GABAA reversal (Buchin et al., 2016; Huberfeld et al., 2011; Lillis et al., 2013), together with an increase in extracellular potassium concentrations (Bazhenov et al., 2004; Krishnan et al., 2011) and decreased activity of interneurons (Ziburkus et al., 2006), all contribute to seizure initiation. Combination of these factors and their relative contribution should be evaluated via further experiments and modeling.

4. The authors choose to call preictal discharges the activity they observe when synaptic inhibition was blocked. One could also interpret this activity as being late spike-and-wave discharges happening towards the end of the seizure. Indeed, the frequency of the discharges tends to decrease towards the end of the seizure, down to 1Hz sometimes. The absence of inhibition would then correspond to the inhibitory neurons being in a depolarization block state. Furthermore, the model only displays such activity if the network is already initialized in the ictal state. Is there any further evidence that this activity resembles more preictal discharges than ictal spike-and-wave discharges?

-&gt; We do not have the feeling that preictal discharges are end-of-seizure persisting (with no end) discharges since they can be generated without any seizure only by increasing excitability (Huberfeld et al. 2011, fig 4d). Further, their pattern is similar before the first seizure (in pro-ictogenic condition with efficient GABAergic signalling) compared to remaining preictal discharges once seizures are blocked by NMDA receptors antagonists (Huberfeld et al. 2011, fig 5b). We have added the following corrections to clarify this point.

Corrections:

Pre-ictal discharges are generated before seizure initiation and in the absence of inhibition when seizures have been established in the slice (Huberfeld et al., 2011). These oscillations are still generated in the absence of inhibitory population (Fig. 2C). Using the model, we show that in this case the background synaptic input to the excitatory population I_E(t) is necessary to generate the periodic pre-ictal oscillations. When I_E(t) is absent, there are no pre-ictal discharges in the model, Fig. 3C. We speculate that before seizure initiation the interneurons are becoming non-functional because of depolarization block (Ziburkus et al., 2006) and GABAA reversal (Lillis et al., 2013) thus allowing the pre-ictal discharges to be generated before seizure initiation (Huberfeld et al., 2011). The proposed model could explain the presence of pre-ictal discharges only in the complete absence of inhibition, Fig. 2C. The possibility of pre-ictal discharge generation before seizure due to non-functional inhibition could be investigated in the future studies.

During seizures or ictal discharges, the frequency content of spiking activity might substantially change (Fig. 3E). This can be explained using the current model as due to the gradual increase of recurrent excitation (Fig. 3A) or the increase of recurrent inhibition. Note that the frequency content of seizure oscillations in the end of it might be similar to the pre-ictal discharges (Fig. 2C.). However, pre-ictal oscillations are possible in the model only in the absence of inhibition (Fig. 2B), as in the experimental data when the GABAA synaptic activity is completely blocked.

5. The abstract mentions that synaptic noise was essential for seizure generation, but it is not clear how that is demonstrated in the text.

-&gt; We have removed the incorrect mention of the synaptic noise from the abstract.

Corrections:

We found that synaptic conductances and slow adaptation in the excitatory population both play essential roles for generating seizures and pre-ictal oscillations.

6. The model appears to have been validated by qualitative comparison of power spectra and time series between simulated and real data. Is there a way to quantify it? My suggestion is to use some basic features (band power, peak frequency, skewness, kurtosis, etc) of your current data that clearly show the distinction between A, B, and C in Figure 2, but show similarity between the model and the real data.

-&gt; We have added the spectrum detailed analysis to the table and provided its description.

Corrections:

For quantitative comparisons between the model and experiment we used the linear fit to the power spectrum over frequencies and peak estimation, as shown in Table 3. We found that there is substantial intersection between linear fits applied to the power spectrums in resting, seizure, and pre-ictal states, as shown in Fig. 2. We found that there is a substantial overlap between these frequencies, providing validation for the model. Note that we compared the overall spectral characteristics between the model and experiment by variation of only one parameter, to reproduce transitions between the pre-ictal, resting and seizure states. If more parameters are varied at the same time, it would be possible to get a better match between the model and experiment.

Minor

1. The introduction should focus more on the novelty of the current paper with regards to excitability, and focus less on the excitation/inhibition balance in past studies (see R1 point #1)

1. the introduction focuses on the balance between excitation and inhibition as the key point in previous studies, but I disagree with that statement. Imbalances were taken into account in almost all models cited, and those give rise to seizure-like activity. I would advise the authors to rather focus on the novelty of their approach, namely the introduction and analysis of excitability, which seems to the Referee to be the key element in their paper and the central novelty compared to the literature.

Corrections:

However, not all types of epileptic seizures can be explained by looking only at the balance between excitation and inhibition (Traub et al., 2005); intrinsic excitability changes on the single-neuron level also play an important role (Krishnan et al., 2011).

Corrections:

These findings motivate the development of neural mass models that can capture the intrinsic excitability in coupled neural populations.

2. Be accurate in descriptions of past models (R1 point #2).

2. Previous models are sometimes described not very accurately. For instance, Touboul's et al model is not based on spiking units, but on a rate model introduced by Jansen & Rit. If the Referee is correct, both papers looked at changes in excitation/inhibition balance and how those changed activity patterns. I would suggest the authors to be more precise in their description.

Corrections:

In neural mass models, neuron populations are often treated as rate units lacking intrinsic adaptation (Touboul et al., 2011). The dynamic behavior of the neural populations is determined by the balance between excitation and inhibition.

3. Computer code should have a simple README to explain what each file does.

Corrections:

README file with the detailed description of the models has been added.

4. Reviewer 2 has a list of minor items below that should be corrected.

The Method section mentions that data from 45 patients were examined. How so? I only see example traces for a couple of patients. Were any parameters fitted to the data of these 45 patients?

-&gt; The model parameters were fitted for one representative patient and corresponding recordings in resting state, seizure state and pre-ictal like oscillations.

Corrections:

The data for the model was taken from one representative patient in the brain slice demonstrating resting state, seizure and pre-ictal oscillations.

Please describe how the power spectrum was computed. The code shows that fft was used. Estimators that address bias/variance issues should be used instead, e.g. multitaper methods (Mitra & Pesaran 2011).

-&gt; FFT is a standard tool to get the power spectrum estimate. Yet we agree that fft might incorporate certain biases into the estimate as mentioned by the reviewer. We have used multitaper power-spectrum estimate in the seizure and pre-ictal states to estimate the major frequency of oscillations, https://ch.mathworks.com/help/signal/ref/pmtm.html.

Corrections:

Power spectrum estimation was performed using fast Fourier transforms. The major frequencies of oscillations were computed via the multitaper method (Thomson 1982).

How were the parameters for the conductances chosen in the first place?

-&gt; Initial parameters were chosen manually based on the available literature data.

Corrections:

The initial parameter set was chosen manually to reproduce the pre-ictal like oscillations due to balance between and, seizure and resting state were fit such that parameter variations would make a transition between seizure and resting state.

-When were the NBQX and d,I-AP5 used? Please describe in the legend of Fig. 2 the conditions used in each experiment, as well as the values of gIE used in the simulations.

Corrections:

-Removed “NBQX and d,I-AP5 were used to block glutamatergic signalling”, the previous statement was not correct. We have used only GABAA blocker.

Corrections:

Corresponding model parameters (A): g_EE=1.5 uS/cm2; g_EI=1 uS/cm2; g_IE=2 uS/cm2; g_II=0.2 uS/cm2; g_AHP=1.6 uS/cm2; (B): g_EE=1.5 uS/cm2; g_EI=1 uS/cm2; g_IE=0.5 uS/cm2; g_II=0.2 uS/cm2; g_AHP=1.6 uS/cm2; (C): g_EE=1.5 uS/cm2; g_EI=1 uS/cm2; g_IE=0 uS/cm2; g_II=0.2 uS/cm2; g_AHP=1.6 uS/cm2.

-What does it mean for the model to be initialized in the seizure state? Are the initial conditions set differently?

-&gt; Initial conditions for variables and parameter values were set in the seizure state.

Corrections:

In these simulations the initial conditions and parameter values of the model were set to the seizure state, but with the conductance (from the inhibitory to the excitatory population) set to zero to mimic the experimental conditions.

For the analysis of gAHP and VGABA, how were the values of gIE chosen? Why wasn't gIE set to 0, as suggested by the experiments?

-&gt; gIE = 0 was suggested for the experiments only for pre-ictal like oscillations, Fig. 2C. We made the following corrections to clarify these points:

Corrections:

To find this regime we slightly modified the parameter set, where g_IE=0.5 mS/cm2 instead of 2 mS/cm2, corresponding to the dynamic regime where becomes the bifurcation parameter.

Corrections:

The analysis was performed for slightly modified parameter set, where =1 mS/cm2, such that becomes the bifurcation parameter. The other parameters remained the same.

Page 10: what are vE, vE/I and vI?

Corrections:

The sigmoid approximation allows one to efficiently take into account zero and linear parts of the potential-to-rate transfer functions, and provides saturation due to the single neuron refractory period (Renart et al., 2004). The sigmoid functions of excitatory and inhibitory populations are shown in Fig. 1C and 1D.

Page 17: gIE should be gEI.

Corrections:

Similar to the g_EE bifurcation diagram, seizure dynamics are possible for a large range of g_EI.

Page 17: please clarify “the increased potassium reversal potential still drives the inhibitory population?” What does drive mean in dynamical terms here?

Corrections:

-&gt; It happens because it decreases the leak current thus depolarizing the membrane potential of excitatory and inhibitory neurons.

- Page 18: I do not see how Fig. 4C demonstrates the assertion “it occasionally brings the system to the oscillatory regime associated with seizure”.

Corrections:

When there is stochastic synaptic input, it occasionally brings the system to the oscillatory regime associated with seizures (Fig. 2C).

- Page 20: Fig. 4E should be Fig. 4F

Corrections:

We found that when V_GABA is higher than -59mV, it leads to ictal oscillations (Fig. 4F).

Fig. 2: the seizures appear to have a hypersynchronous onset (as opposed to a low-voltage fast activity onset). Does that mean this model is restricted to this type of seizure? If so, please mention it.

Corrections:

We compared the model power spectrum with the measured LFP recorded during the initial phase of the ictal discharge with the hypersynchronous activity onset.

- Legend of Fig. 2: describe the blue and red traces. The left plot title should be “Power spectrum”, not the name of the state. Use different colours for LFP and intracellular recordings and describe them in the legend.

Corrections:

Red traces correspond to the model, blue traces to the experiment. Green traces are the intracellular recordings from the pyramidal cells.

Corrections:

-&gt; Added green marker for the intracellular recordings and “power spectrum” label.

-Fig. 4A: shouldn't the “Rest” label be for low values of gEE?

Corrections:

-&gt; Added second “Rest” label to all figures:

Fig. 4: does the stable fixed point on the other side of the seizure correspond to saturation of the model?

Yes, it corresponds to the saturation of the transfer function.

Corrections:

When self-excitation is further increased up to 4 mS/cm2, seizure-related oscillations disappear since the system moves to the high activity state due to sigmoidal saturation of the transfer function, as shown in Fig. 1C and 1D.

Table 1: references for Ureset and Vthreshold?

Table 1: typo: Interpretation for gIE: conductance

-&gt; Corrected

Table 2: I(t) should be IE(t)

-&gt; corrected

INa, IK, ICl and the firing rate are not described in Table 2.

-&gt; INa, IK, ICl are dependent variables, therefore are not listed in Table 2.

Bartolomei et al. (2014) and Proix et al. (2014) refer to the same paper. I would also suggest citing work Ullah et al. (2009) and Cressman et al. (2009).

Corrections:

To describe the additional mechanisms of seizure transition, the present model could include slow activity-dependent parameter changes similar to (Proix et al., 2014, Bartolomei et al., 2014; Ullah et al., 2009; Cressman et al., 2009).

## References

[B2] Bazhenov M, Timofeev I, Steriade M, Sejnowski TJ (2004) Potassium model for slow (2-3 Hz) in vivo neocortical paroxysmal oscillations. J Neurophysiol 92:1116–1132. 10.1152/jn.00529.2003 15056684 PMC2925854

[B3] Beghi E, Berg A, Carpio A, Forsgren L, Hesdorffer DC, Hauser WA, Malmgren K, S S, Temkin N, Thurman D, Tomson T (2005) Comment on epileptic seizures and epilepsy: definitions proposed by the International League Against Epilepsy (ILAE) and the International Bureau for Epilepsy (IBE). Epilep 46:1698–1699. 10.1111/j.1528-1167.2005.00273_1.x. 16190948

[B4] Brunel N, Wang XJ (2001) Effects of neuromodulation in a cortical network model of object working memory dominated by recurrent inhibition. J Comput Neurosci 11:63–85. [10.1007/s10827-014-0506-8] 11524578

[B5] Buchin A, Chizhov AV (2010a) Modified firing-rate model reproduces synchronization of a neuronal population receiving complex input. Opt Mem Neural Netw 19:166–171. 10.3103/S1060992X10020074

[B6] Buchin A, Chizhov A (2010b) Firing-rate model of a population of adaptive neurons. Biofizika 55:592–599. 10.1134/S0006350910040135 20968079

[B7] Buchin A, Rieubland S, Häusser M, Gutkin BS, Roth A (2016a) Inverse stochastic resonance in cerebellar purkinje cells. PLoS Comput Biol 12:e1005000. 10.1371/journal.pcbi.1005000 27541958 PMC4991839

[B8] Buchin A, Chizhov A, Huberfeld G, Miles R, Gutkin BS (2016b) Reduced efficacy of the KCC2 cotransporter promotes epileptic oscillations in a subiculum network model. J Neurosci 36:11619–11633. 10.1523/JNEUROSCI.4228-15.2016 27852771 PMC6231544

[B9] Buzsáki G, Anastassiou CA, Koch C (2012) The origin of extracellular fields and currents - EEG, ECoG, LFP and spikes. Nat Rev Neurosci 13:407–420. 10.1038/nrn3241 22595786 PMC4907333

[B10] Chin JH, Vora N (2014) The global burden of neurologic diseases. Neurology 83:349–351. 10.1212/WNL.0000000000000610 25049303 PMC4115599

[B11] Chizhov AV (2002) Model of evoked activity of populations of neurons in the hippocampus. Biofizika 47:1007–1015. 12500573

[B12] Chizhov AV, Graham L (2007) Population model of hippocampal pyramidal neurons, linking a refractory density approach to conductance-based neurons. Phys Rev E Stat Nonlin Soft Matter Phys 75:011924 10.1103/PhysRevE.75.011924 17358201

[B13] Chizhov AV, Rodrigues S, Terry JR (2007) A comparative analysis of an EEG model and a conductance-based neural population model. Phys Let A 369:31–36. 10.1016/j.physleta.2007.04.060

[B14] Chizhov AV, Graham LJ (2008) Efficient evaluation of neuron populations receiving colored-noise current based on a refractory density method. Phys Rev E Stat Nonlin Soft Matter Phys 77:011910 10.1103/PhysRevE.77.011910 18351879

[B15] Chizhov AV (2014) Conductance-based refractory density model of primary visual cortex. J Comput Neurosci 36:297–319. 10.1007/s10827-013-0473-5 23888313

[B16] Chizhov AV, Sanchez-Aguilera A, Rodrigues S, de la Prida LM (2015) Simplest relationship between local field potential and intracellular signals in layered neural tissue. Phys Rev E Stat Nonlin Soft Matter Phys 92:062704 10.1103/PhysRevE.92.062704 26764724

[B17] Chizhov AV, Amakhin DV, Zaitsev AV (2017) Computational model of interictal discharges triggered by interneurons. PLoS One 12:0185752 10.1371/journal.pone.0185752 PMC5627938 28977038

[B18] Chizhov AV, Zefirov AV, Amakhin DV, Smirnova EY, Zaitsev AV (2018) Minimal model of interictal and ictal discharges “Epileptor-2.” PLoS Comput Biol 14:e1006186. 10.1371/journal.pcbi.1006186 29851959 PMC6005638

[B19] Cohen I, Navarro V, Clemenceau S, Baulac M, Miles R (2002) On the origin of interictal activity in human temporal lobe epilepsy in vitro. Science 298:1418–1421. 10.1126/science.1076510 12434059

[B20] Cressman JR Jr, Ullah G, Ziburkus J, Schiff SJ, Barreto E (2009) The influence of sodium and potassium dynamics on excitability, seizures, and the stability of persistent states: I. Single neuron dynamics. J Comput Neurosci 26:159–170. 10.1007/s10827-008-0132-4 19169801 PMC2704057

[B21] de la Prida LM, Huberfeld G, Cohen I, Miles R (2006) Threshold behavior in the initiation of hippocampal population bursts. Neuron 49:131–142. 10.1016/j.neuron.2005.10.034 16387645

[B22] Demont-Guignard S, Benquet P, Gerber U, Wendling F (2009) Analysis of intracerebral EEG recordings of epileptic spikes: insights from a neural network model. IEEE Trans Biomed Eng 56:2782–2795. 10.1109/TBME.2009.2028015 19651549 PMC3245744

[B23] Dietzel I, Heinemann U (1986) Dynamic variations of the brain cell microenvironment in relation to neuronal hyperactivity. Ann NY Acad Sci 481:72–84. 3468867 10.1111/j.1749-6632.1986.tb27140.x

[B24] Engel J, Bragin A, Staba R, Mody I (2009) High-frequency oscillations: what is normal and what is not? Epilepsia 50:598–604. 10.1111/j.1528-1167.2008.01917.x 19055491

[B25] Florence G, Dahlem MA, Almeida ACG, Bassani JWM, Kurths J (2009) The role of extracellular potassium dynamics in the different stages of ictal bursting and spreading depression: a computational study. J Theor Biol 258:219–228. 10.1016/j.jtbi.2009.01.032 19490858

[B26] Fröhlich F, Bazhenov M (2006) Coexistence of tonic firing and bursting in cortical neurons. Phys Rev E Stat Nonlin Soft Matter Phys 74:031922 10.1103/PhysRevE.74.031922 17025682

[B27] Fröhlich F, Bazhenov M, Iragui-Madoz V, Sejnowski TJ (2008) Potassium dynamics in the epileptic cortex: new insights on an old topic. Neuroscientist 14:422–433. 10.1177/1073858408317955 18997121 PMC2854295

[B28] Gerstner W, Kistler W (2002) Spiking neuron models: single neurons, populations, plasticity. Cambridge, UK: Cambridge University Press.

[B29] Hall D, Kuhlmann D (2013) Mechanisms of seizure propagation in 2-dimensional centre-surround recurrent networks. PLoS One 8:e71369 10.1371/journal.pone.0071369 23967201 PMC3742758

[B30] Huberfeld G, Wittner L, Clemenceau S, Baulac M, Kaila K, Miles R, Rivera C (2007) Perturbed chloride homeostasis and GABAergic signaling in human temporal lobe epilepsy. J Neurosci 27:9866–9873. 10.1523/JNEUROSCI.2761-07.2007 17855601 PMC6672644

[B31] Huberfeld G, de la Prida ML, Pallud J, Cohen I, Le Van Quyen M, Adam C, Clemenceau S, Baulac M, Miles R (2011) Glutamatergic pre-ictal discharges emerge at the transition to seizure in human epilepsy. Nat Neurosci 14:627–634. 10.1038/nn.2790 21460834

[B32] Jansen BH, Rit VG (1995) Electroencephalogram and visual evoked potential generation in a mathematical model of coupled cortical columns. Biol Cybern 73:357–366. 10.1007/BF00199471 7578475

[B33] Jedlicka P, Deller T, Gutkin BS, Backus KH (2011) Activity-dependent intracellular chloride accumulation and diffusion controls GABA(A) receptor-mediated synaptic transmission. Hippocampus 21:885–898. 20575006 10.1002/hipo.20804

[B34] Jensen MS, Azouz R, Yaari Y (1994) Variant firing patterns in rat hippocampal pyramidal cells modulated by extracellular potassium. J Neurophysiol 71:831–839. 10.1152/jn.1994.71.3.831 8201423

[B35] Jirsa V, Sporns O, Breakspear M, Deco G, McIntosh AR (2010) Towards the virtual brain: network modeling of the intact and the damaged brain. Arch Ital de Biol 148(3):189–205. 21175008

[B36] Jirsa VK, Stacey WC, Quilichini PP, Ivanov AI, Bernard C (2014) On the nature of seizure dynamics. Brain 137:2210–2230. 10.1093/brain/awu133 24919973 PMC4107736

[B37] Johannesma PIM (1968) Diffusion models for the stochastic activity of neurons In: Neural Networks, pp 116–144. Berlin: Springer Verlag.

[B38] Jung HY, Staff NP, Spruston N (2001) Action potential bursting in subicular pyramidal neurons is driven by a calcium tail current. J Neurosci 21:3312–3321. 11331360 10.1523/JNEUROSCI.21-10-03312.2001 PMC6762486

[B39] Kager H, Wadman WJ, Somjen GG (2000) Simulated seizures and spreading depression in a neuron model incorporating interstitial space and ion concentrations. J Neurophysiol 84:495–512. 10.1152/jn.2000.84.1.495 10899222

[B40] Karnup S, Stelzer A (1999) Temporal overlap of excitatory and inhibitory afferent input in guinea-pig CA1 pyramidal cells. J Physiol 516:485–504. 10.1111/j.1469-7793.1999.0485v.x 10087347 PMC2269280

[B41] Karus C, Mondragão MA, Ziemens D, Rose CR (2015) Astrocytes restrict discharge duration and neuronal sodium loads during recurrent network activity. Glia 63:936–957. 10.1002/glia.22793 25639699

[B42] Krishnan GP, Bazhenov M (2011) Ionic dynamics mediate spontaneous termination of seizures and postictal depression state. J Neurosci 31:8870–8882. 10.1523/JNEUROSCI.6200-10.2011 21677171 PMC3163257

[B43] Lillis KP, Kramer MA, Mertz J, Staley KJ, White JA (2012) Pyramidal cells accumulate chloride at seizure onset. Neurobiol Dis 47:358–366. 10.1016/j.nbd.2012.05.016 22677032 PMC3392473

[B44] Lytton WW (2008) Computer modelling of epilepsy. Nat Rev Neurosci 9:626–637. 10.1038/nrn2416 18594562 PMC2739976

[B45] Marder E, Taylor AL (2011) Multiple models to capture the variability in biological neurons and networks. Nat Neurosci 14:133–138. 10.1038/nn.2735 21270780 PMC3686573

[B46] Molaee-Ardekani B, Benquet P, Bartolomei F, Wendling F (2010) Computational modeling of high-frequency oscillations at the onset of neocortical partial seizures: from “altered structure” to “dysfunction.” Neuroimage 52:1109–1122. 10.1016/j.neuroimage.2009.12.049 20034581

[B47] Naze S, Bernard C, Jirsa V (2015) Computational modeling of seizure dynamics using coupled neuronal networks: factors shaping epileptiform activity. PLoS Comput Biol 11:e1004209 10.1371/journal.pcbi.1004209 25970348 PMC4430284

[B48] Pallud J, Le van Quyen M, Bielle F, Pellegrino C, Varlet P, Labussiere M, Cresto N, Baulac M, Duyckaerts C, Kourdougli N, Chazal G, Devaux B, Rivera C, Miles R, Capelle L, Huberfeld G (2014) Cortical GABAergic excitation contributes to epileptic activities around human glioma. Sci Transl Med 6:244ra89 10.1126/scitranslmed.3008065 PMC4409113 25009229

[B49] Pinsky PF, Rinzel J (1994) Intrinsic and network rhythmogenesis in a reduced Traub model for CA3 neurons. J Comput Neurosci 1:39–60. 10.1007/BF00962717 8792224

[B50] Prinz A, Bucher D, Marder E (2004) Similar network activity from disparate circuit parameters. Nat Neurosci 7:1345–1352. 10.1038/nn1352 15558066

[B51] Proix T, Bartolomei F, Chauvel P, Bernard C, Jirsa VK (2014) Permittivity coupling across brain regions determines seizure recruitment in partial epilepsy. J Neurosci 34:15009–15021. 10.1523/JNEUROSCI.1570-14.2014 25378166 PMC6608363

[B52] Quilichini PP, Le Van Quyen M, Ivanov A, Turner DA, Carabalona A, Gozlan H, Esclapez M, Bernard C (2012) Hub GABA neurons mediate gamma-frequency oscillations at ictal-like event onset in the immature hippocampus. Neuron 74:57–64. 10.1016/j.neuron.2012.01.026 22500630 PMC3328133

[B53] Ratnadurai-Giridharan S, Stefanescu RA, Khargonekar PP, Carney PR, Talathi SS (2014) Genesis of interictal spikes in the CA1: a computational investigation. Front Neural Circuits 8:2 10.3389/fncir.2014.00002 24478636 PMC3902301

[B54] Renart A, Brunel N, Wang XJ (2004) Mean field theory of irregularly spiking neuronal populations and working memory in recurrent cortical networks In: Computational neuroscience: a comprehensive approach, pp 425–463. Boca Raton, FL: CRC Press.

[B55] Rothstein JD, Patel S, Regan MR, Haenggeli C, Huang YH, Bergles DE, Jin L, Hoberg MD, Vidensky S, Chung DS, Toan SV, Bruijn LI, Su ZZ, Gupta P, Fisher PB (2005) Beta-lactam antibiotics offer neuroprotection by increasing glutamate transporter expression. Nature 433:73–77. 10.1038/nature03180 15635412

[B56] Izhikevich EM (2007) Dynamical systems in neuroscience. Cambridge, MA: MIT Press.

[B57] Sivakumaran S, Cardarelli RA, Maguire J, Kelley MR, Silayeva L, Morrow DH, Mukherjee J, Moore YE, Mather RJ, Duggan ME, Brandon NJ, Dunlop J, Zicha S, Moss SJ, Deeb TZ (2015) Selective inhibition of KCC2 leads to hyperexcitability and epileptiform discharges in hippocampal slices and in vivo. J Neurosci 35:8291–8296. 10.1523/JNEUROSCI.5205-14.2015 26019342 PMC4444547

[B58] Stiefel KM, Gutkin BS, Sejnowski TJ (2008) Cholinergic neuromodulation changes phase response curve shape and type in cortical pyramidal neurons. PLoS One 3:e3947. 10.1371/journal.pone.0003947 19079601 PMC2596483

[B59] Thomson DJ (1982) Spectrum estimation and harmonic analysis. Proc IEEE 70:1055–1096. 10.1109/PROC.1982.12433

[B60] Touboul J, Wendling F, Chauvel P, Faugeras O (2011) Neural mass activity, bifurcations, and epilepsy. Neural Comput 23:3232–3286. 10.1162/NECO_a_00206 21919787

[B61] Traub RD, Contreras D, Whittington MA (2005) Combined experimental/simulation studies of cellular and network mechanisms of epileptogenesis in vitro and in vivo. J Clin Neurophysiol 22:330–342. 16357637

[B62] Ullah G, Cressman JF Jr, Barreto E, Schiff JS (2009) The influence of sodium and potassium dynamics on excitability, seizures, and the stability of persistent states: II. Network and glial dynamics. J Comput Neurosci 26:171–183. 10.1007/s10827-008-0132-4 19083088 PMC2951284

[B63] Ursino M, la Cara GE (2006) Travelling waves and EEG patterns during epileptic seizure: analysis with an integrate-and-fire neural network. J Theor Biol 242:171–187. 10.1016/j.jtbi.2006.02.012 16620870

[B64] Wendling F, Bartolomei F, Bellanger JJ, Chauvel P (2002) Epileptic fast activity can be explained by a model of impaired GABAergic dendritic inhibition. Eur J Neurosci 15:1499–1508. 10.1046/j.1460-9568.2002.01985.x 12028360

[B65] Wendling F, Bartolomei F, Mina F, Huneau C, Benquet P (2012) Interictal spikes, fast ripples and seizures in partial epilepsies-combining multi-level computational models with experimental data. Eur J Neurosci 36:2164–2177. 10.1111/j.1460-9568.2012.08039.x 22805062

[B66] Wilson HR, Cowan JD (1972) Excitatory and inhibitory interactions in localized populations of model neurons. Biophys J 12:1–24. 10.1016/S0006-3495(72)86068-5 4332108 PMC1484078

[B67] Xiong ZQ, Stringer JL (1999) Astrocytic regulation of the recovery of extracellular potassium after seizures in vivo. Eur J Neurosci 11:1677–1684. 10215921 10.1046/j.1460-9568.1999.00587.x

[B68] Ziburkus J, Cressman JR, Barreto E, Schiff SJ (2006) Interneuron and pyramidal cell interplay during in vitro seizure-like events. J Neurophysiol 95:3948–3954. 10.1152/jn.01378.2005 16554499 PMC1469233

