# Supplementary material for: Adaptation and Inhibition Control Pathological Synchronization in a Model of Focal Epileptic Seizure
Source: eNeuro. 2018 Oct 5;5(5):ENEURO.0019-18.2018. doi: 10.1523/ENEURO.0019-18.2018 (PMC6173584; doi:10.1523/ENEURO.0019-18.2018)
Supplement: Extended Data 1 — The code is available as Extended Data. Download Extended Data, ZIP file [file sup_enu-eN-NWR-0019-18-s03.zip › Github/README.docx]

**README**

This code simulates the E-I population model in XPPAUT. This code allows to generate the time series of the population activity in the excitatory and inhibitory cells during seizure and towards transition to seizure. Some initial ideas for this project were developed during the Advanced Course in Computational Neuroscience (<http://www.neuroinf.pl/accn>).

Here is the pre-print of the article describing the use of this model:

<https://www.biorxiv.org/content/early/2018/05/02/312561>

**Dependencies:**

-To run XPPAUT code, generate simulaitons and analyse bifurcation diagrams you need to have it installed: <http://www.math.pitt.edu/~bard/xpp/xpp.html>

-Matlab is required for the data analysis: <https://www.mathworks.com/products/matlab.html>

**Instructions:**

Different folders contain the corresponding figures from the papers. To reproduce the simulations from Fig. 2 you need to run the EI_Cress_pop_AHP_bif_noise.ode file in XPPAUT. The parameter sets for resting state, seizure and pre-ictal discharges states are present in corresponding jpg files.

To perform the spectrum analysis the UE variable, corresponding to the activity of the excitatory population should be exported into the dat file. Then the corresponding time and voltage vectors should be imported to Matlab and saved as t, UE variables. Then running the spectrum_timeseries_processing.m would generate the required spectrum.

To reproduce the bifurcation diagrams, it is necessary to open the EI_Cress_pop_AHP_bif_NO_noise.ode in XPPAUT. The file contains the model dynamics in the absence of noise. The equations should be integrated in XPPAUT for at least 5 seconds of time. Then the AUTO package could be started to compute the corresponding one-parameter bifurcations diagrams for gEE, gEI, gIE, gII, gAHP and VGABA parameters. Each subfolder in Fig. 4 folder contains saved bifurcation diagrams after computations in AUTO and XPPAUT. To plot the bifurcation diagrams one could use bif_plot.m in Matlab once corresponding variables are imported.

Once the bifurcation diagrams are computed similar to Fig. 4, one could change the diagrams properties by going to Graphics -> Frequencies. The frequencies of oscillations will be shown, Fig. 3.

In order to generate the power spectrum from the time series one would need to go to power_spectrum folder, open Erest.fig, run the data_extract.m script. Then execute plot_spectrum.m file. This file contains the multitaper spectrum fit function.

In order to fit the power spectrum and evaluate the fit in Matlab it is necessary to type cftool and open sfit file in the corresponding spectrum_fit folder in Fig_2 folder.

Copyright (C) 2018 Anatoly Buchin This program comes with ABSOLUTELY NO WARRANTY. This is free software, and you are welcome to redistribute it under certain conditions.
