# Supplementary figures and images for: Adaptation and Inhibition Control Pathological Synchronization in a Model of Focal Epileptic Seizure
Source: eNeuro. 2018 Oct 5;5(5):ENEURO.0019-18.2018. doi: 10.1523/ENEURO.0019-18.2018 (PMC6173584; doi:10.1523/ENEURO.0019-18.2018)

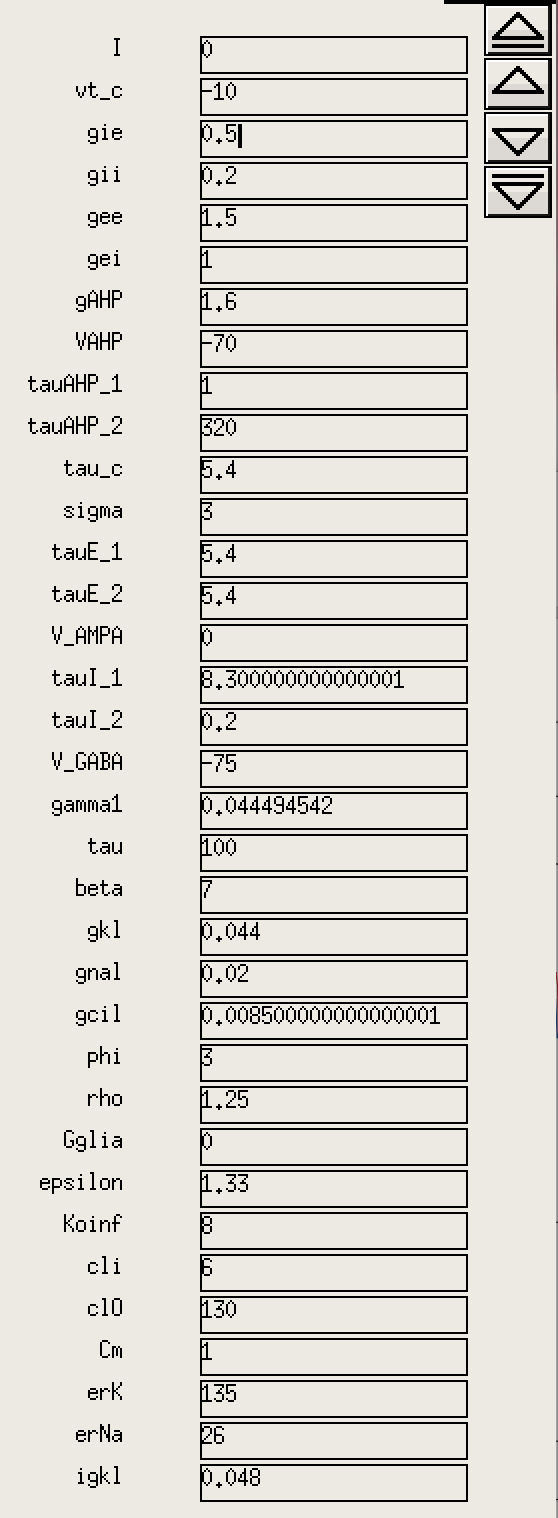

Supplement: Extended Data 1 — The code is available as Extended Data. Download Extended Data, ZIP file [file sup_enu-eN-NWR-0019-18-s03.zip › Github/Fig_2/seizure.png]

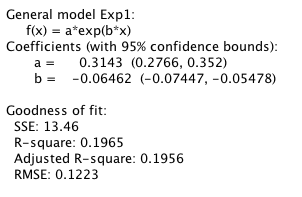

Supplement: Extended Data 1 — The code is available as Extended Data. Download Extended Data, ZIP file [file sup_enu-eN-NWR-0019-18-s03.zip › Github/Fig_2/A - Rest/spectrum_fit/exponential.png]

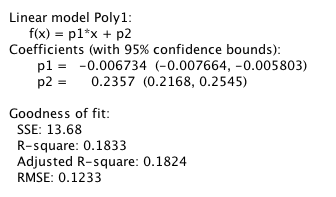

Supplement: Extended Data 1 — The code is available as Extended Data. Download Extended Data, ZIP file [file sup_enu-eN-NWR-0019-18-s03.zip › Github/Fig_2/A - Rest/spectrum_fit/linear_fit.png]

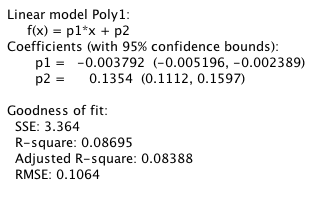

Supplement: Extended Data 1 — The code is available as Extended Data. Download Extended Data, ZIP file [file sup_enu-eN-NWR-0019-18-s03.zip › Github/Fig_2/A - Rest/spectrum_fit/model/linear_model_fit.png]

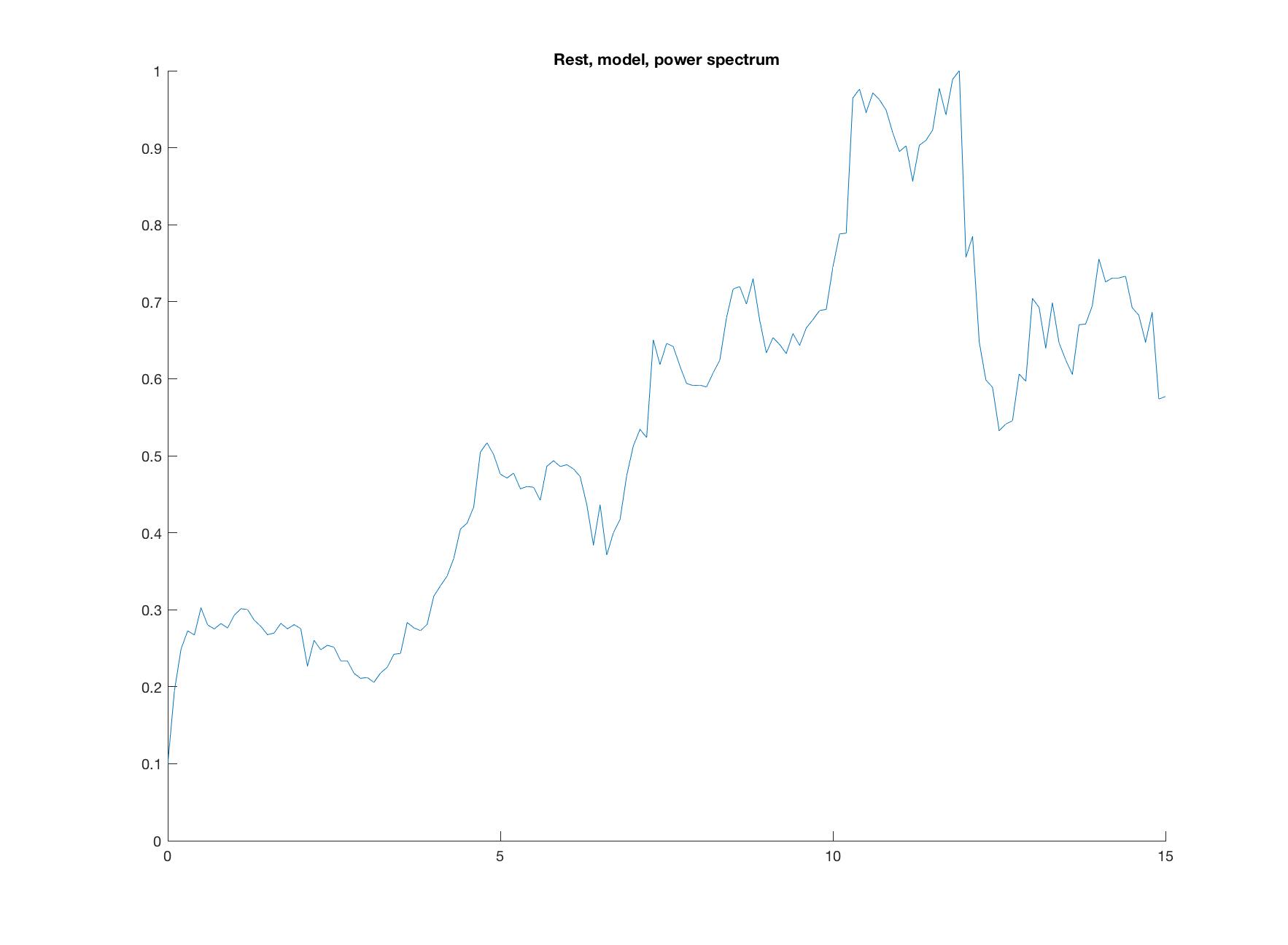

Supplement: Extended Data 1 — The code is available as Extended Data. Download Extended Data, ZIP file [file sup_enu-eN-NWR-0019-18-s03.zip › Github/Fig_2/A - Rest/power_spectrum/spectrum_rest.jpg]

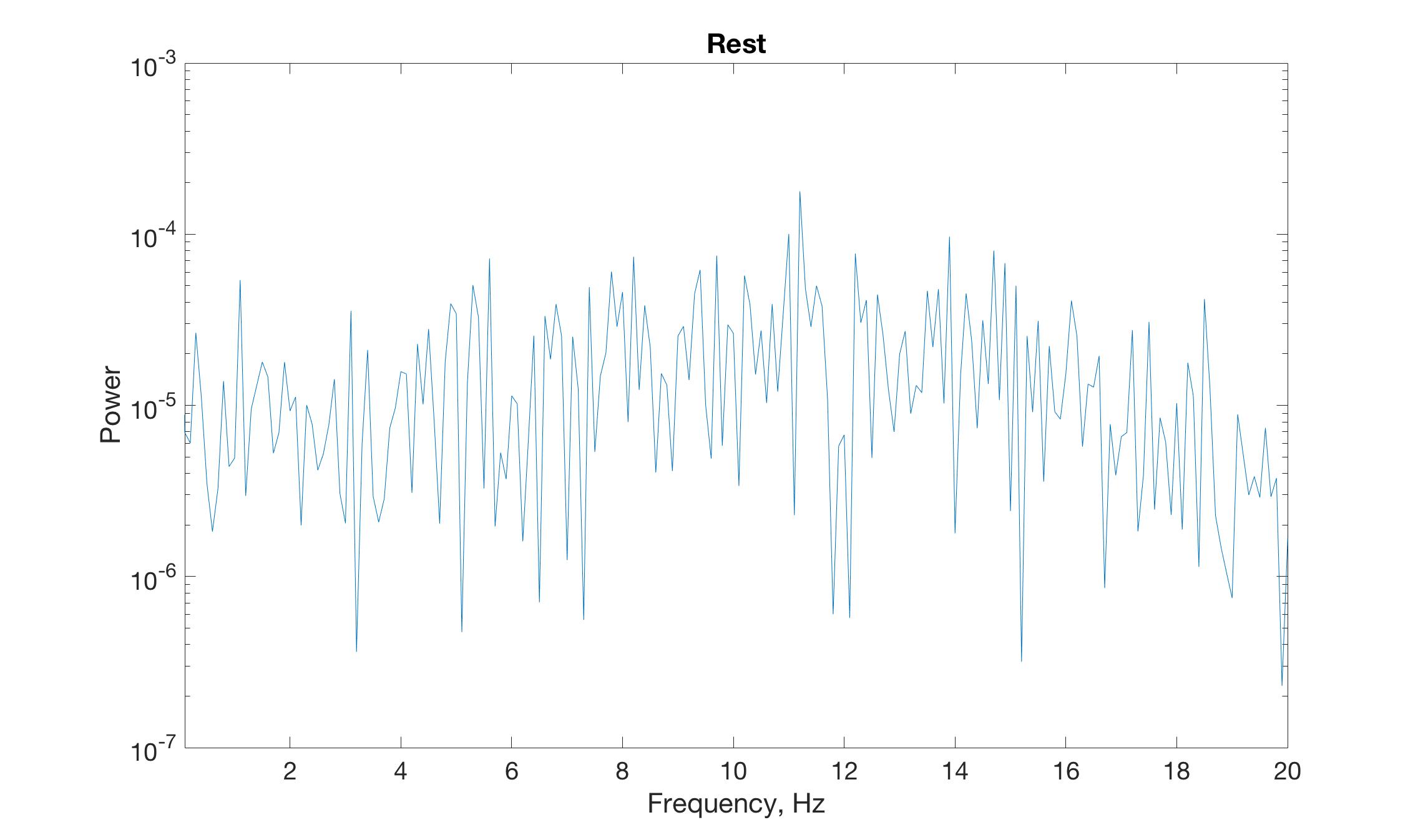

Supplement: Extended Data 1 — The code is available as Extended Data. Download Extended Data, ZIP file [file sup_enu-eN-NWR-0019-18-s03.zip › Github/Fig_2/A - Rest/Rest_spect.jpg]

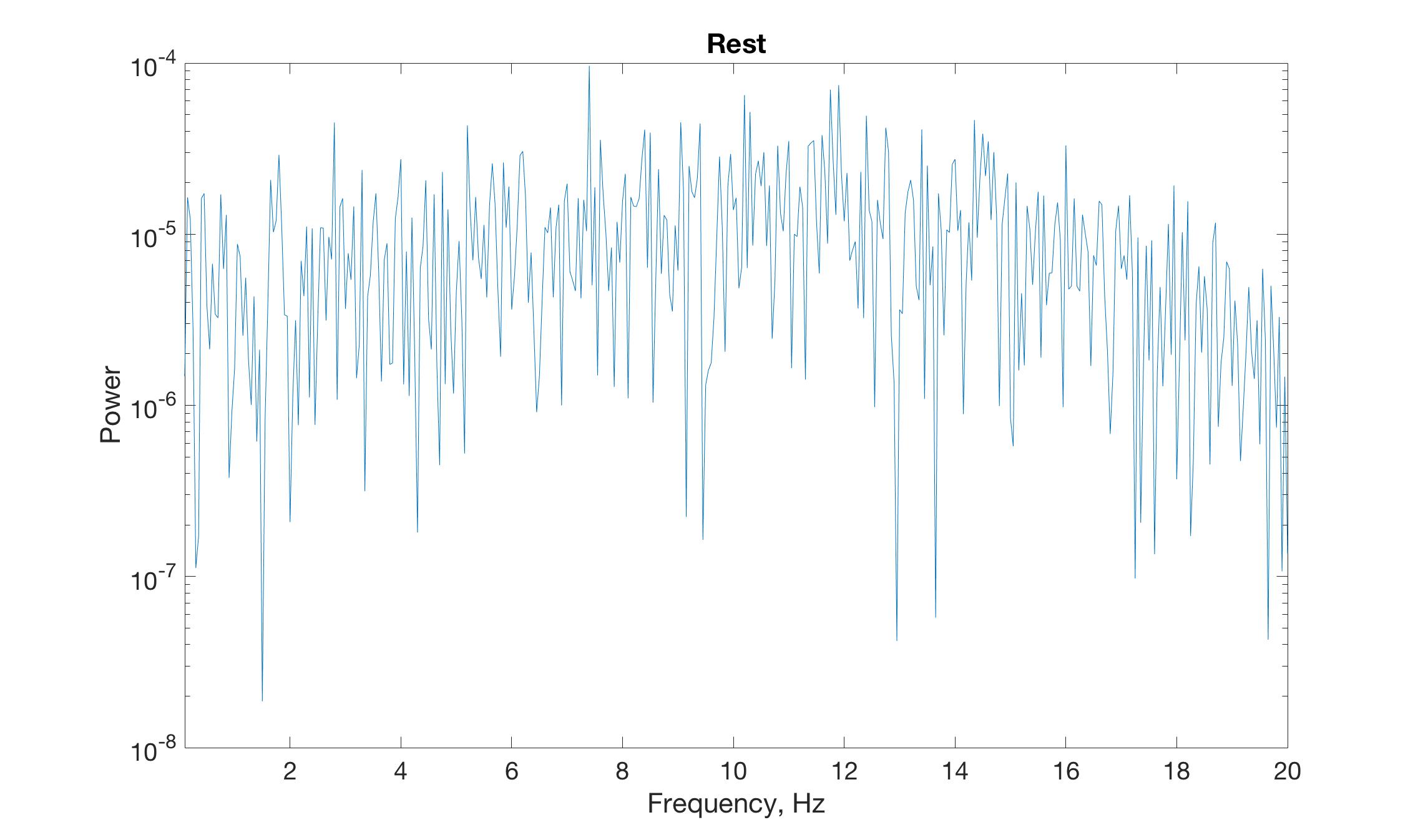

Supplement: Extended Data 1 — The code is available as Extended Data. Download Extended Data, ZIP file [file sup_enu-eN-NWR-0019-18-s03.zip › Github/Fig_2/A - Rest/Rest_spect_long.jpg]

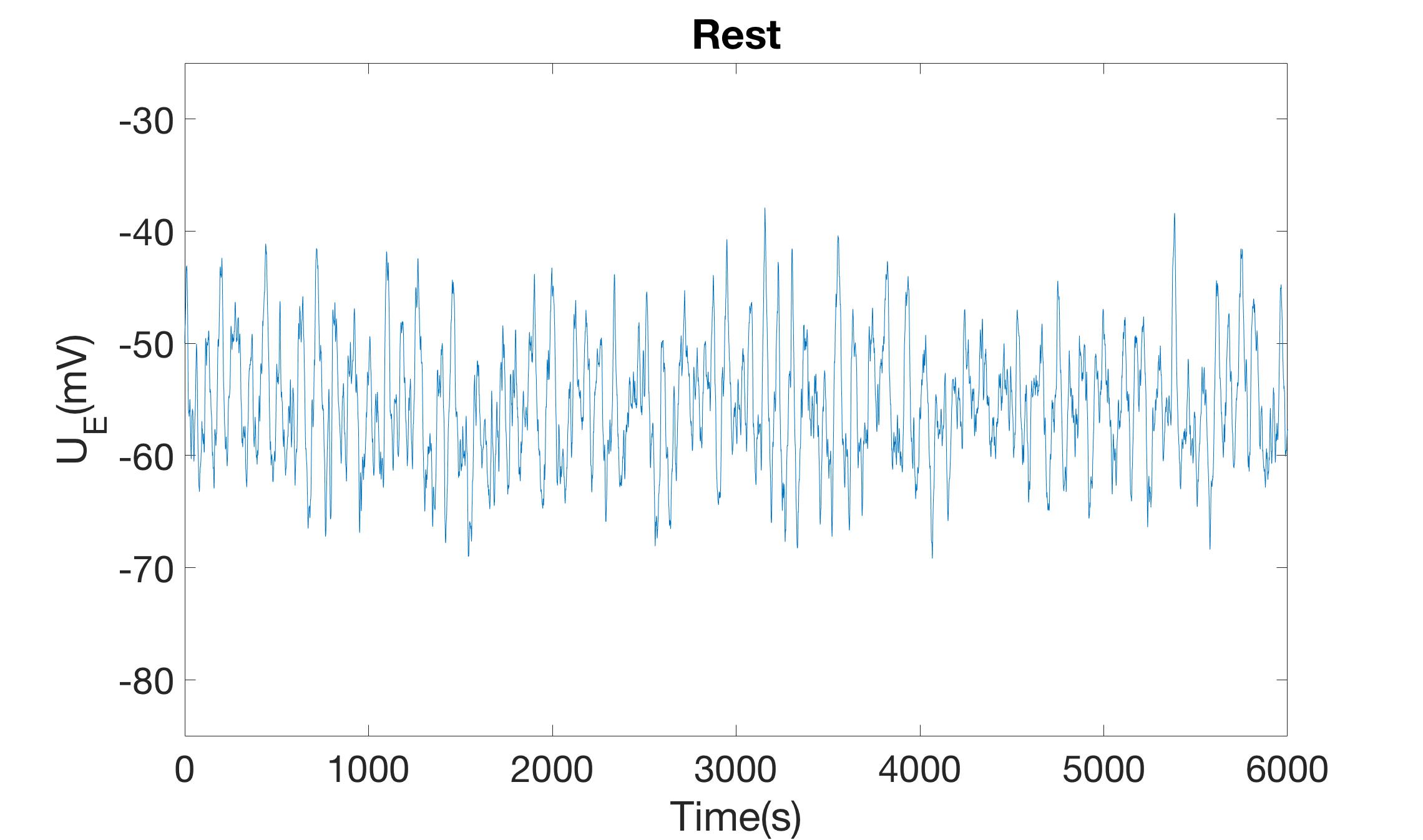

Supplement: Extended Data 1 — The code is available as Extended Data. Download Extended Data, ZIP file [file sup_enu-eN-NWR-0019-18-s03.zip › Github/Fig_2/A - Rest/Rest_time.jpg]

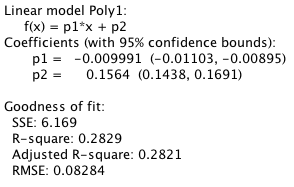

Supplement: Extended Data 1 — The code is available as Extended Data. Download Extended Data, ZIP file [file sup_enu-eN-NWR-0019-18-s03.zip › Github/Fig_2/C - PIDs/spectrum_fit/linear_fit.png]

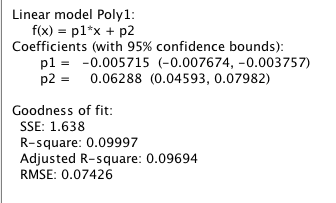

Supplement: Extended Data 1 — The code is available as Extended Data. Download Extended Data, ZIP file [file sup_enu-eN-NWR-0019-18-s03.zip › Github/Fig_2/C - PIDs/spectrum_fit/model/linear_model_fit.png]

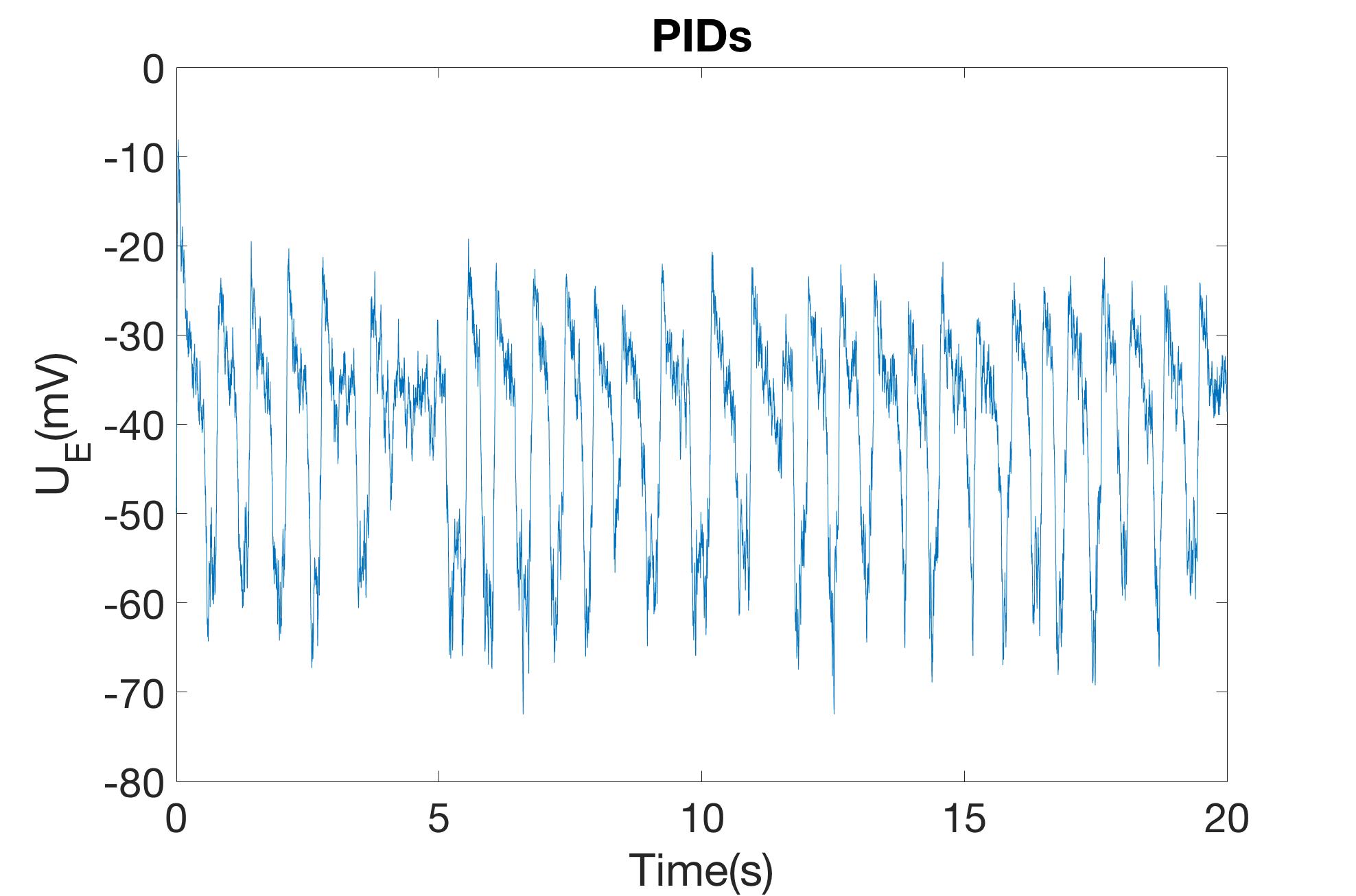

Supplement: Extended Data 1 — The code is available as Extended Data. Download Extended Data, ZIP file [file sup_enu-eN-NWR-0019-18-s03.zip › Github/Fig_2/C - PIDs/PIDs_time.jpg]

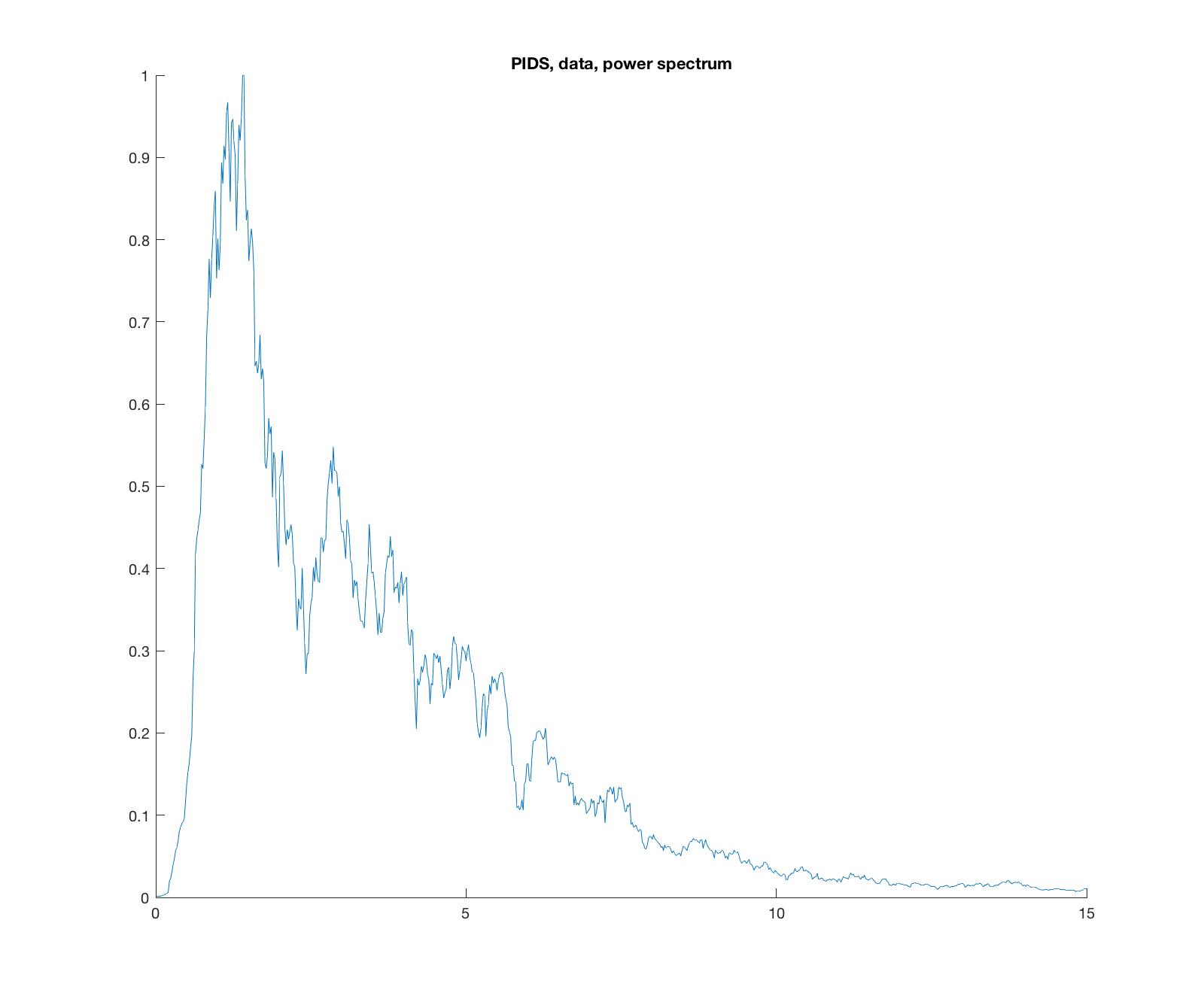

Supplement: Extended Data 1 — The code is available as Extended Data. Download Extended Data, ZIP file [file sup_enu-eN-NWR-0019-18-s03.zip › Github/Fig_2/C - PIDs/power_spectrum/pids_spectrum.jpg]

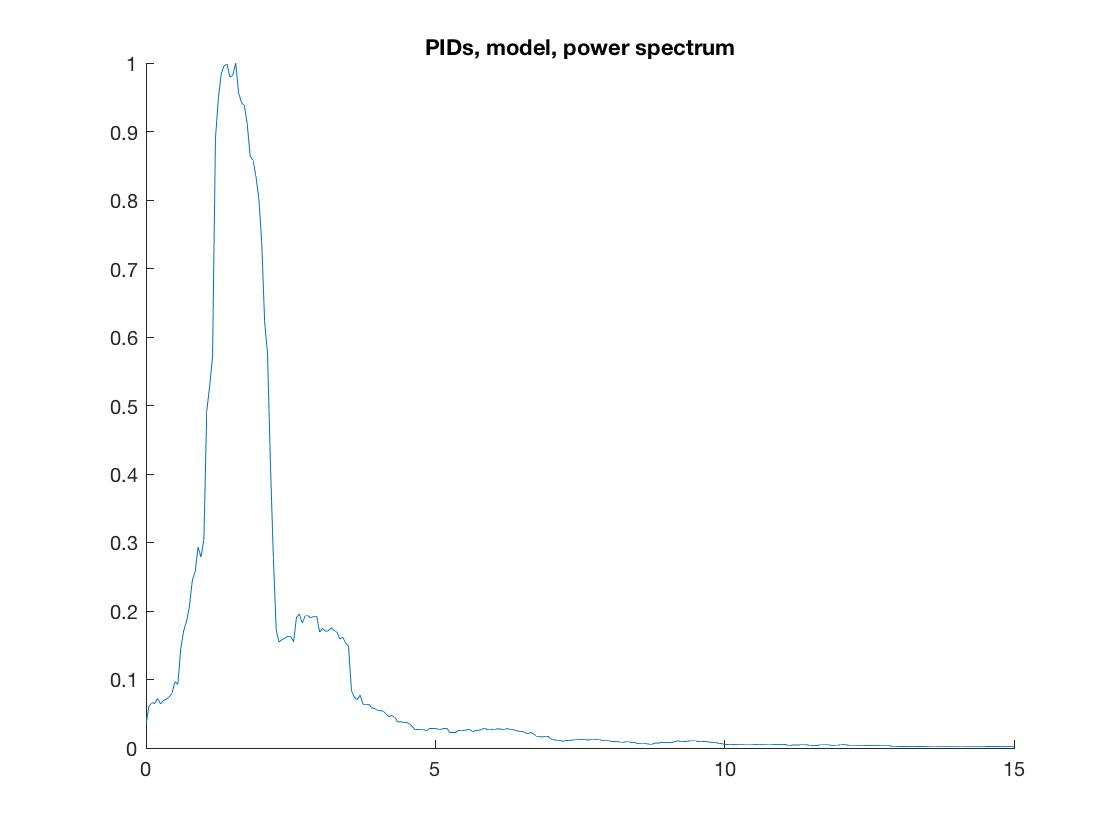

Supplement: Extended Data 1 — The code is available as Extended Data. Download Extended Data, ZIP file [file sup_enu-eN-NWR-0019-18-s03.zip › Github/Fig_2/C - PIDs/power_spectrum/spectrum_PIDs.jpg]

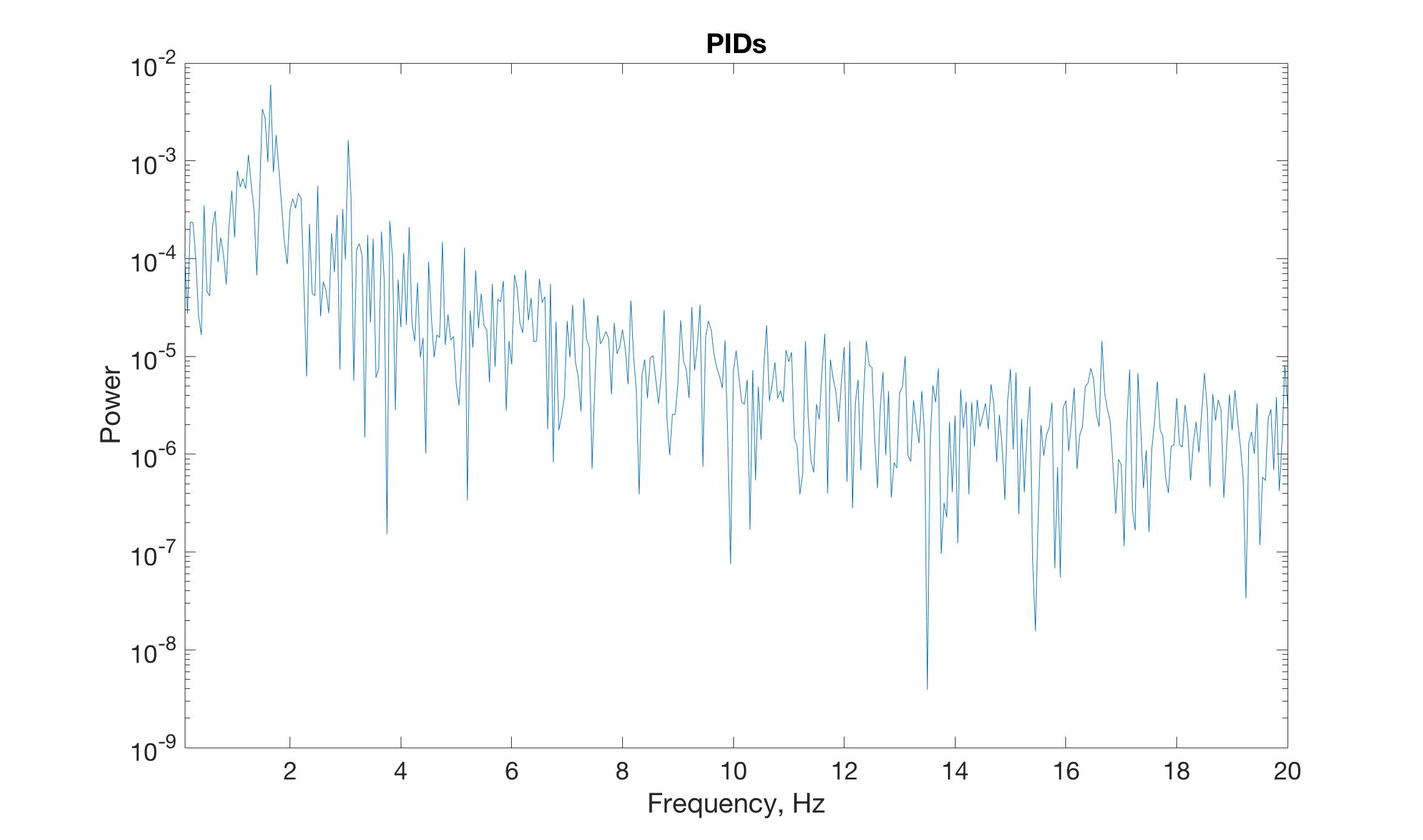

Supplement: Extended Data 1 — The code is available as Extended Data. Download Extended Data, ZIP file [file sup_enu-eN-NWR-0019-18-s03.zip › Github/Fig_2/C - PIDs/PIDs_spect.jpg]

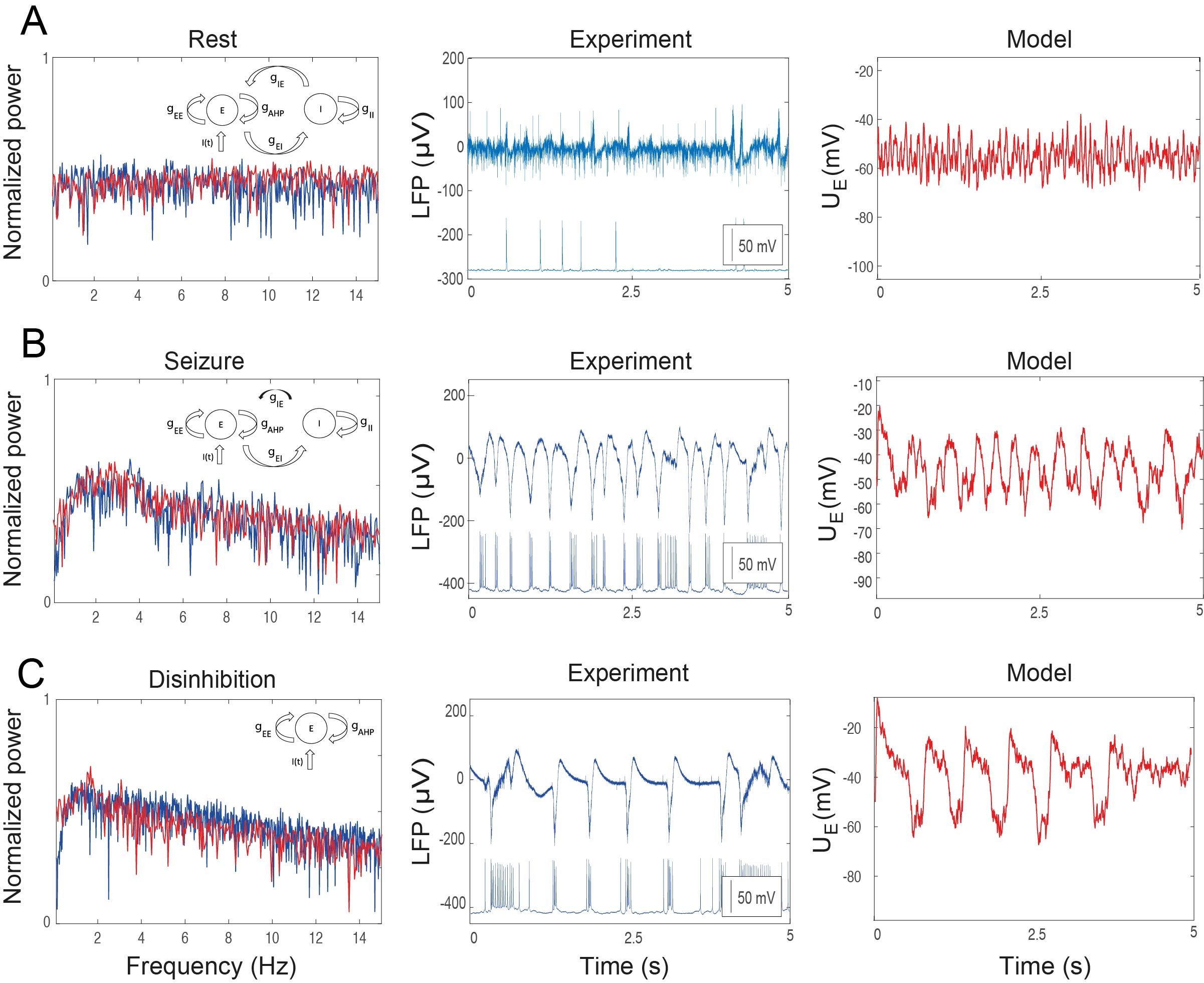

Supplement: Extended Data 1 — The code is available as Extended Data. Download Extended Data, ZIP file [file sup_enu-eN-NWR-0019-18-s03.zip › Github/Fig_2/Fig_2_ver4.jpg]

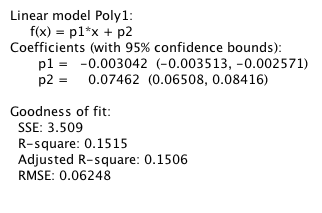

Supplement: Extended Data 1 — The code is available as Extended Data. Download Extended Data, ZIP file [file sup_enu-eN-NWR-0019-18-s03.zip › Github/Fig_2/B - Seizure/spectrum_fit/linear_fit.png]

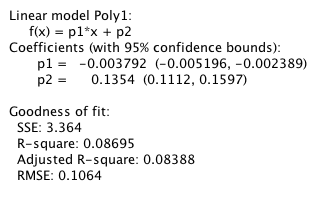

Supplement: Extended Data 1 — The code is available as Extended Data. Download Extended Data, ZIP file [file sup_enu-eN-NWR-0019-18-s03.zip › Github/Fig_2/B - Seizure/spectrum_fit/model/linear_model_fit.png]

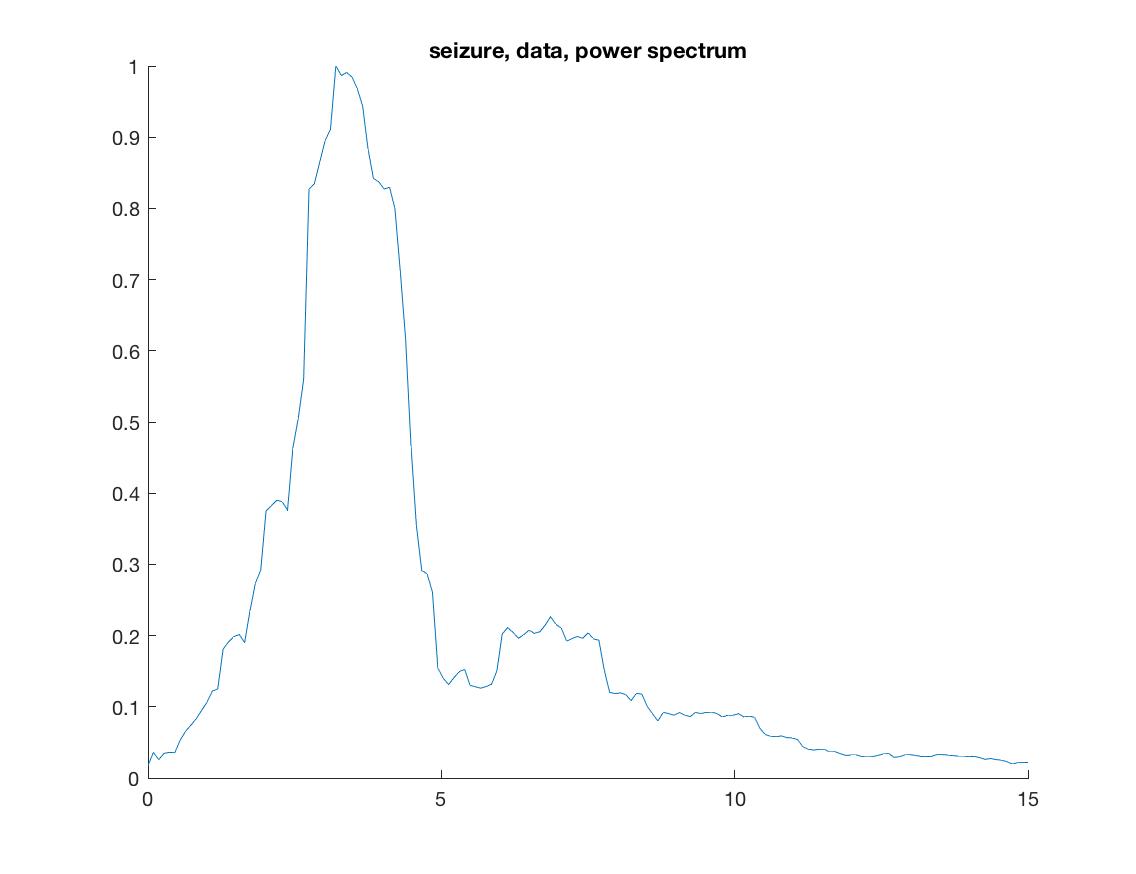

Supplement: Extended Data 1 — The code is available as Extended Data. Download Extended Data, ZIP file [file sup_enu-eN-NWR-0019-18-s03.zip › Github/Fig_2/B - Seizure/power_spectrum/seizure_spectrum.jpg]

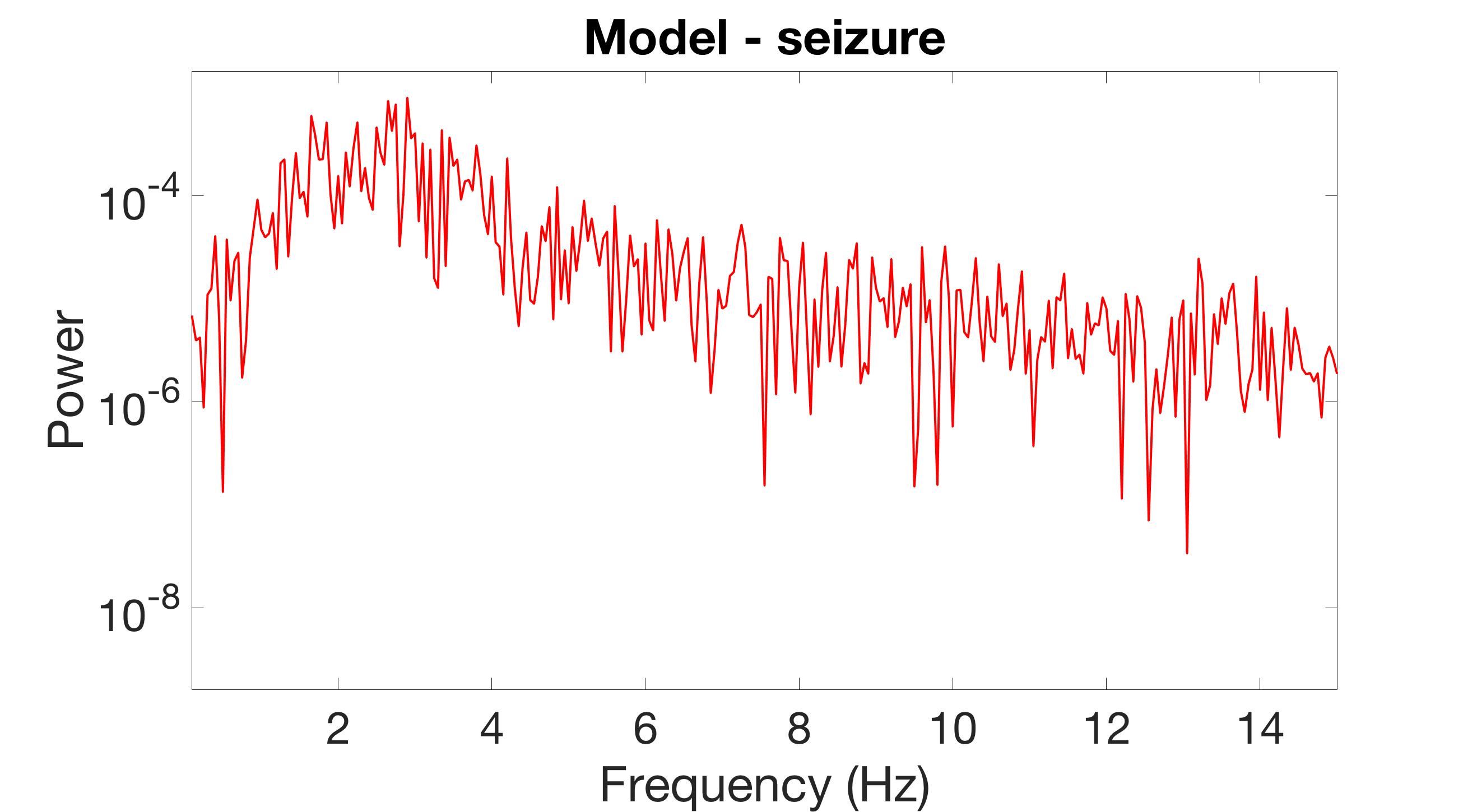

Supplement: Extended Data 1 — The code is available as Extended Data. Download Extended Data, ZIP file [file sup_enu-eN-NWR-0019-18-s03.zip › Github/Fig_2/B - Seizure/Seizure_spect.jpg]

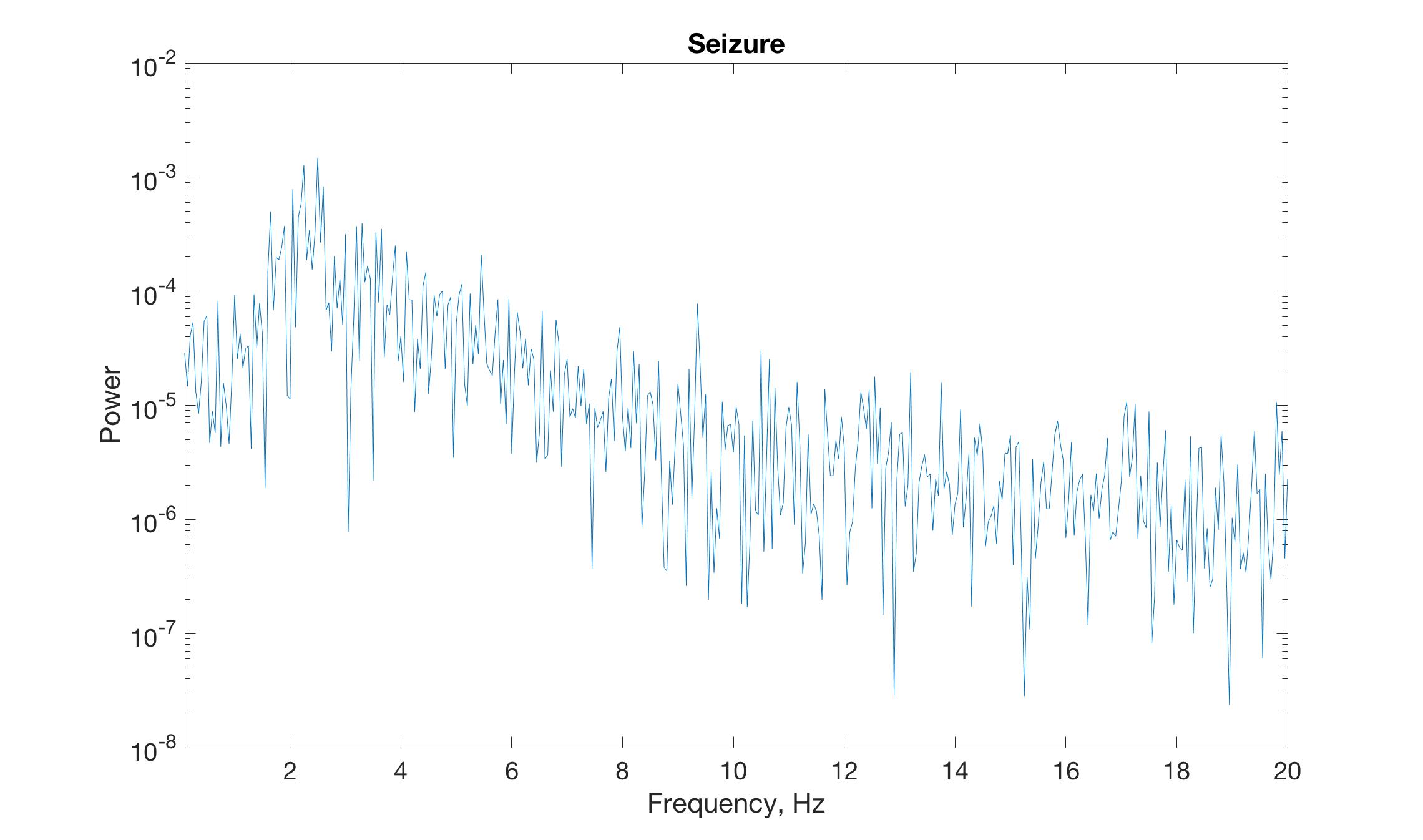

Supplement: Extended Data 1 — The code is available as Extended Data. Download Extended Data, ZIP file [file sup_enu-eN-NWR-0019-18-s03.zip › Github/Fig_2/B - Seizure/Seizure_spect_long.jpg]

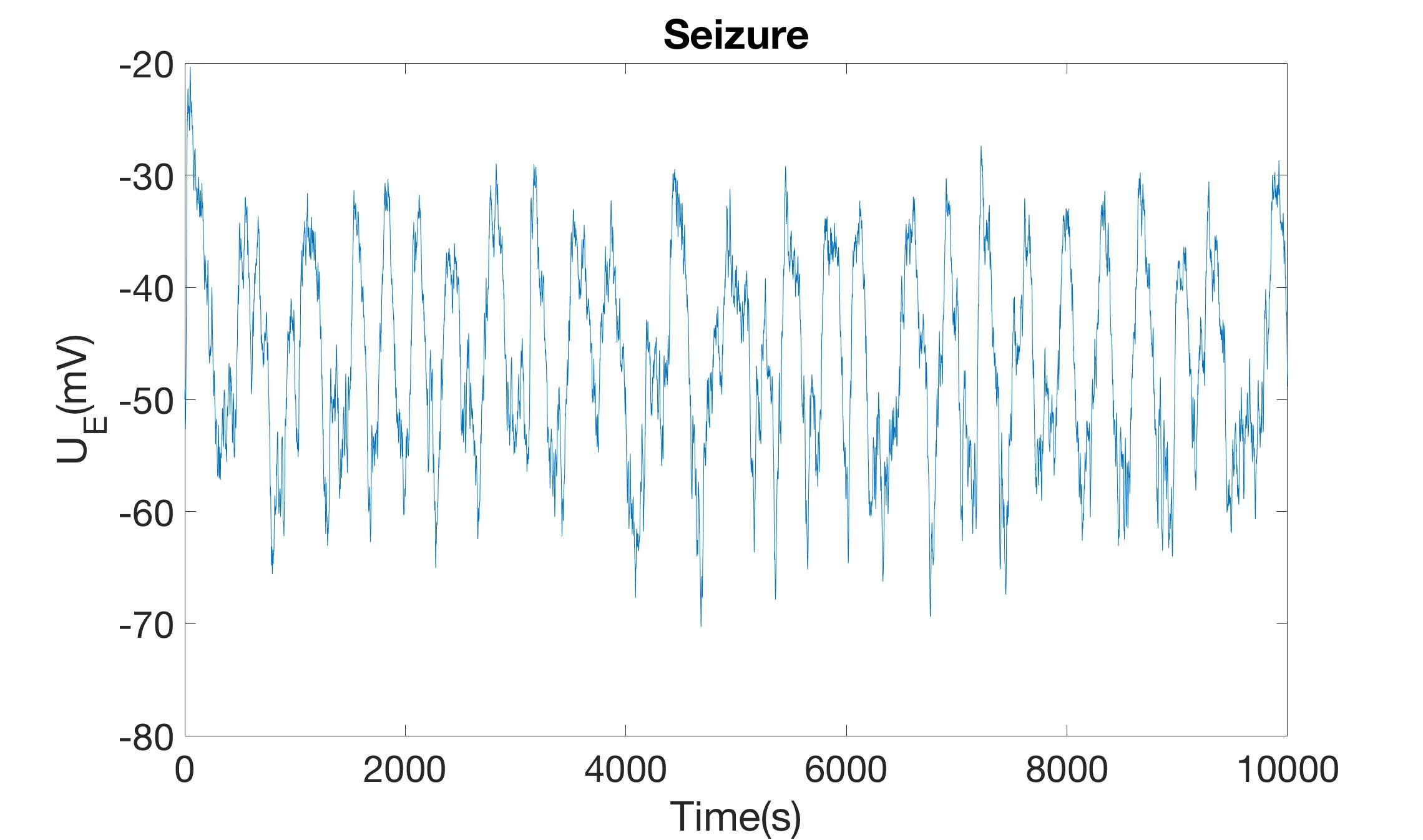

Supplement: Extended Data 1 — The code is available as Extended Data. Download Extended Data, ZIP file [file sup_enu-eN-NWR-0019-18-s03.zip › Github/Fig_2/B - Seizure/Seizure_time.jpg]

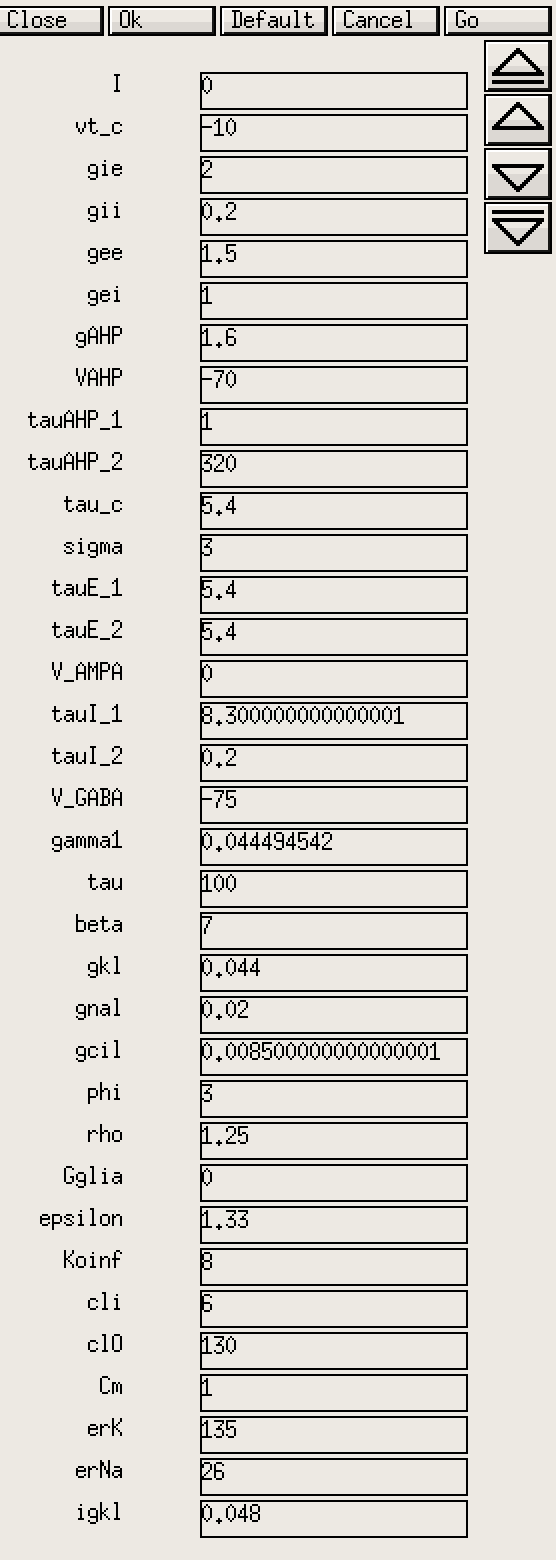

Supplement: Extended Data 1 — The code is available as Extended Data. Download Extended Data, ZIP file [file sup_enu-eN-NWR-0019-18-s03.zip › Github/Fig_2/rest.png]

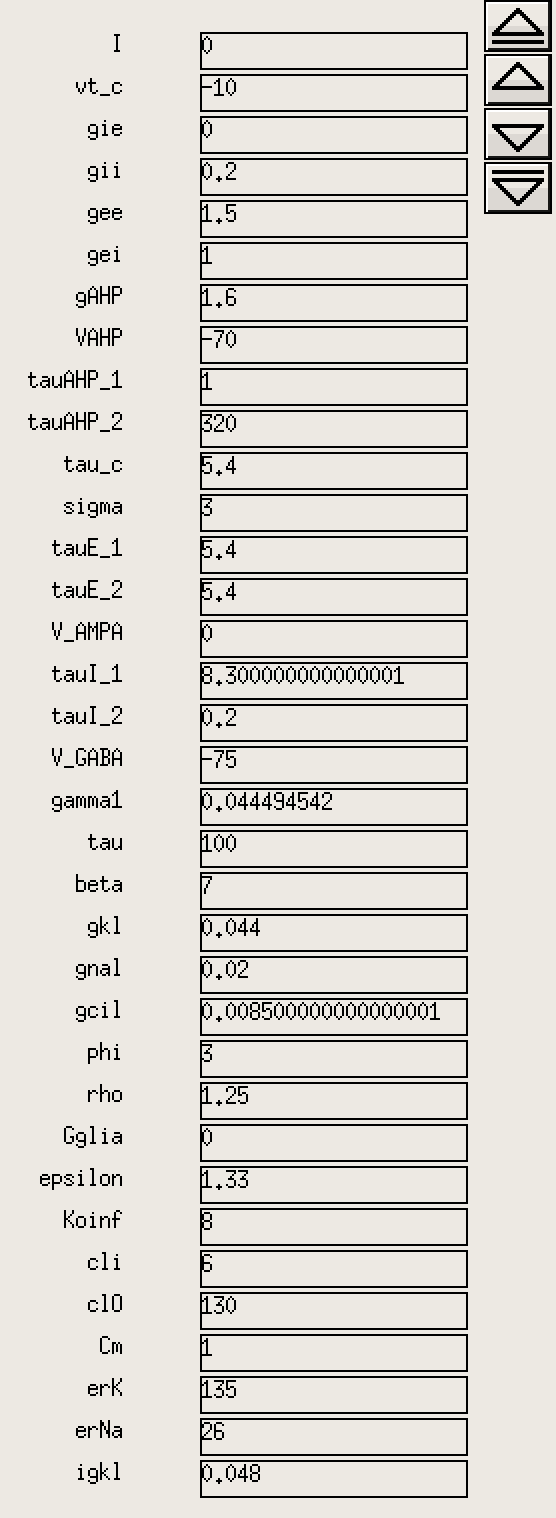

Supplement: Extended Data 1 — The code is available as Extended Data. Download Extended Data, ZIP file [file sup_enu-eN-NWR-0019-18-s03.zip › Github/Fig_2/PIDs.png]

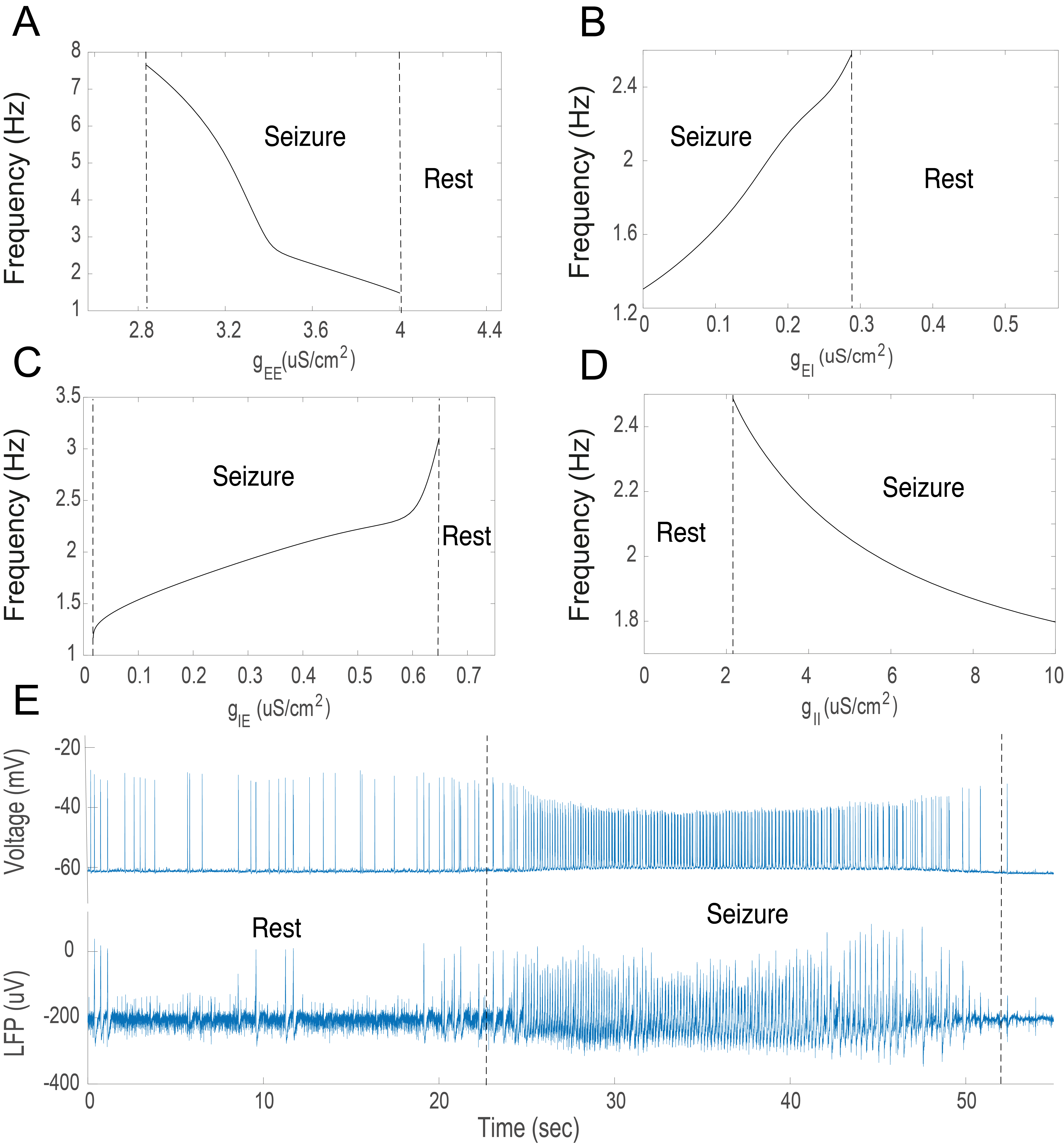

Supplement: Extended Data 1 — The code is available as Extended Data. Download Extended Data, ZIP file [file sup_enu-eN-NWR-0019-18-s03.zip › Github/Fig_3/Fig_3a_newPar.png]

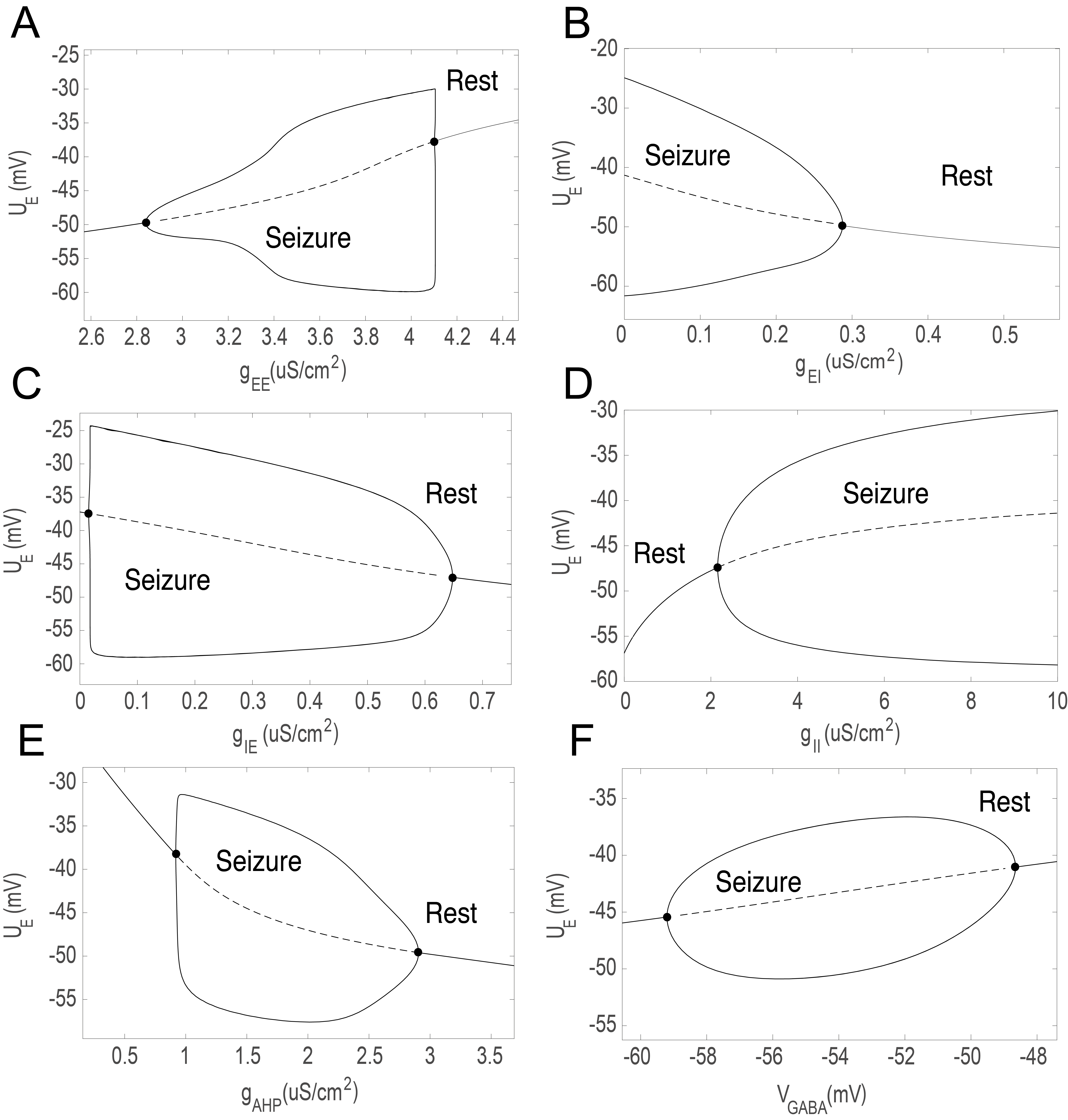

Supplement: Extended Data 1 — The code is available as Extended Data. Download Extended Data, ZIP file [file sup_enu-eN-NWR-0019-18-s03.zip › Github/Fig_4/Fig_3_newPar.png]

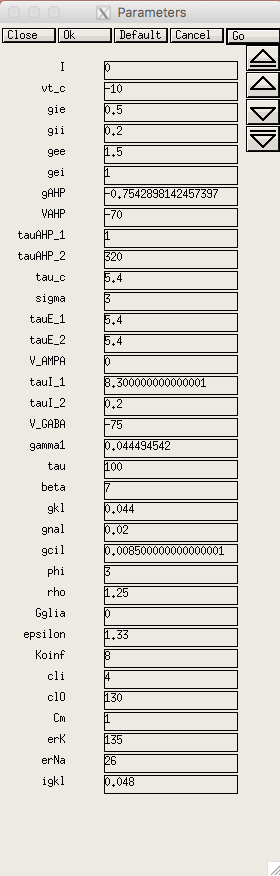

Supplement: Extended Data 1 — The code is available as Extended Data. Download Extended Data, ZIP file [file sup_enu-eN-NWR-0019-18-s03.zip › Github/Fig_4/gAHP/seizure_par.png]

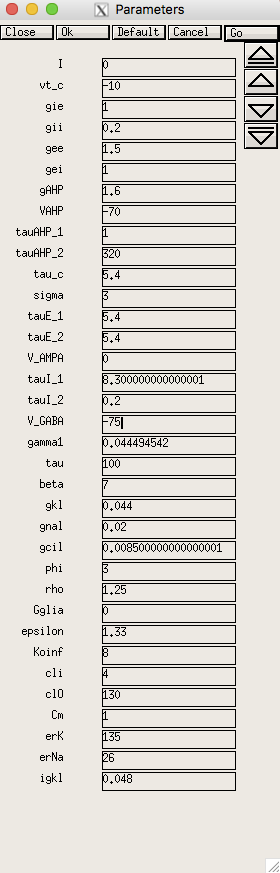

Supplement: Extended Data 1 — The code is available as Extended Data. Download Extended Data, ZIP file [file sup_enu-eN-NWR-0019-18-s03.zip › Github/Fig_4/VGABA/VGABA_par_point.png]

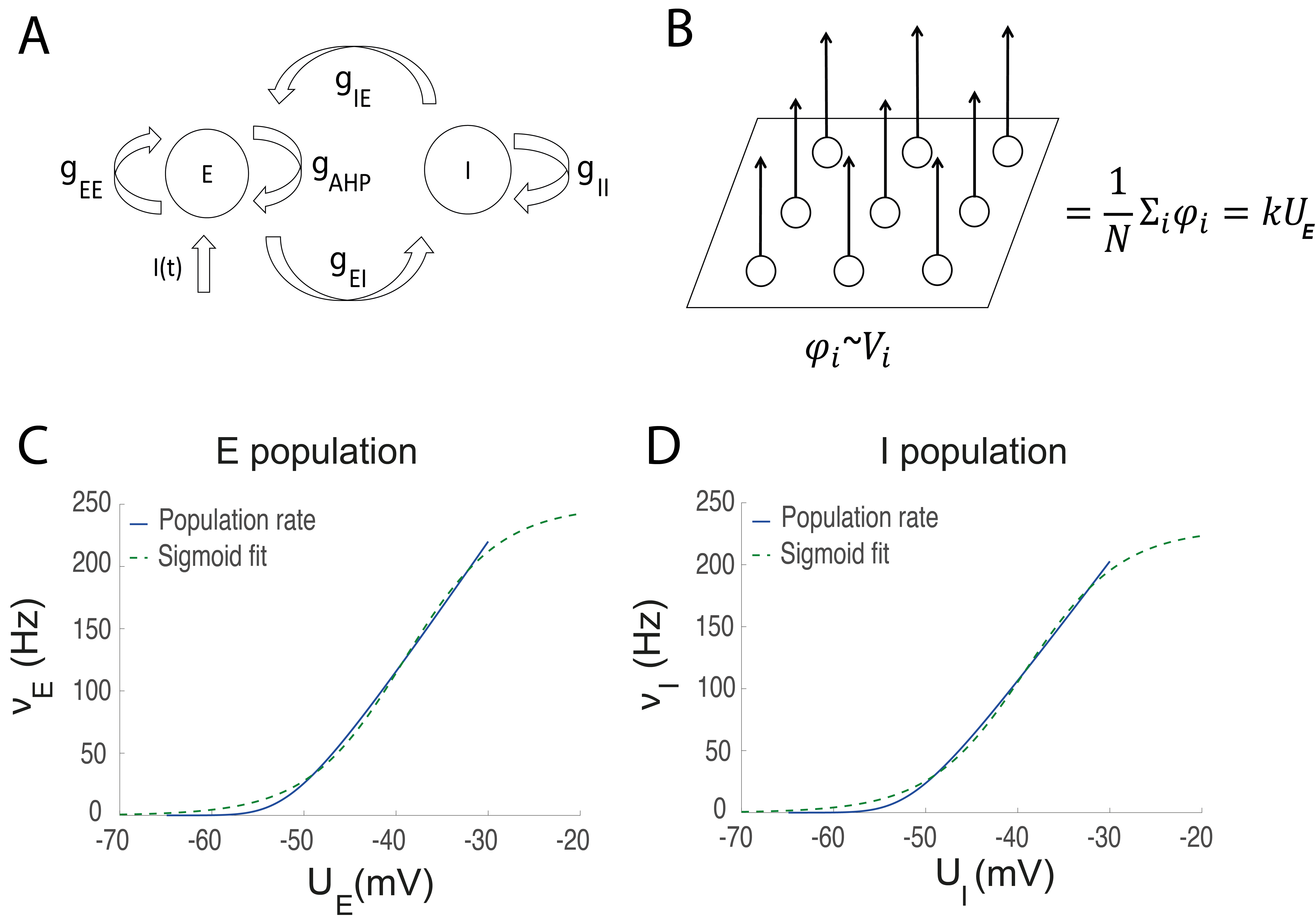

Supplement: Extended Data 1 — The code is available as Extended Data. Download Extended Data, ZIP file [file sup_enu-eN-NWR-0019-18-s03.zip › Github/Fig_1/Fig_1_ver1.png]

## Slide 1
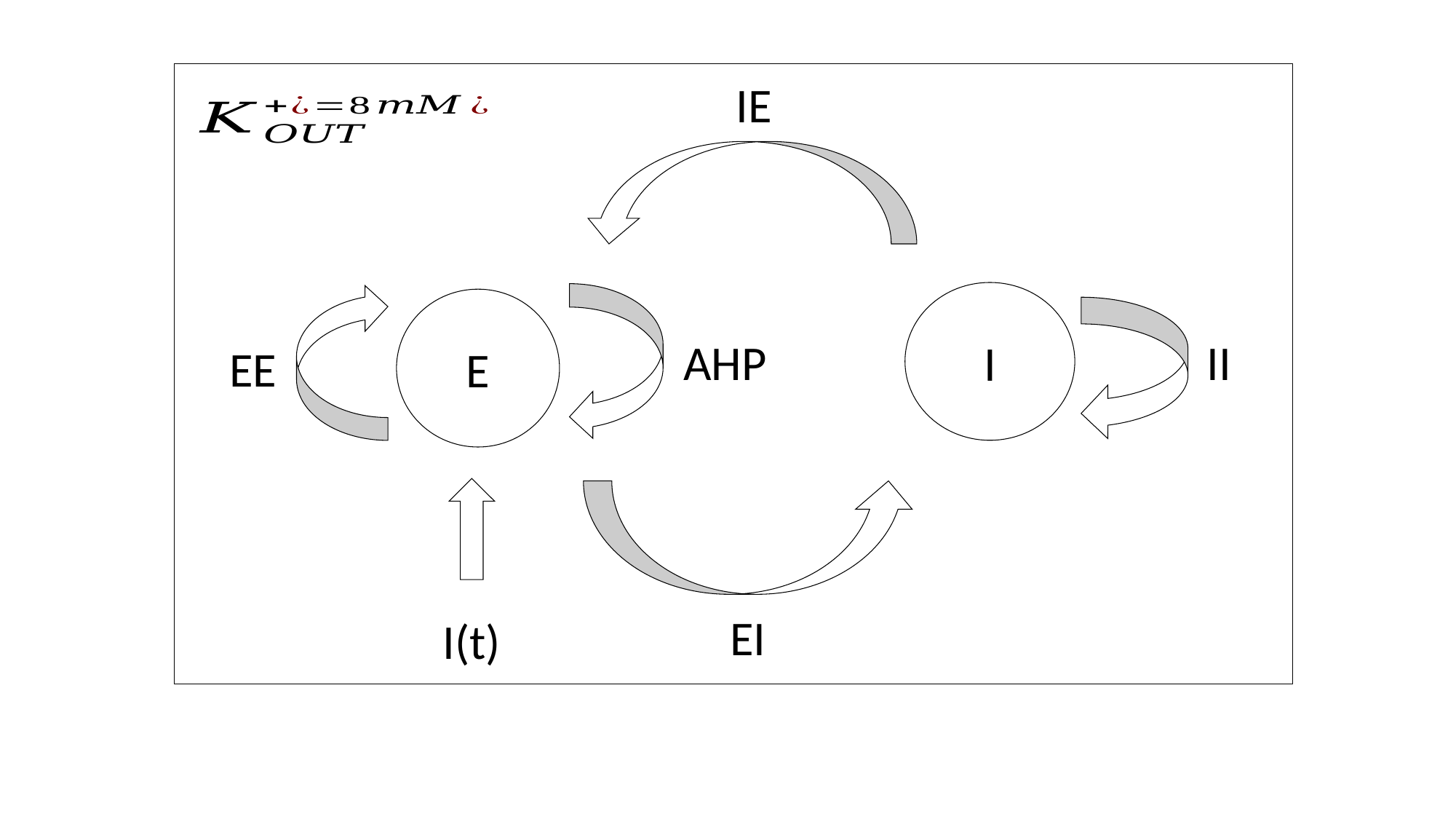

IE
I
E
AHP
II
EE
EI
I(t)

Supplement: Extended Data 1 — The code is available as Extended Data. Download Extended Data, ZIP file [file sup_enu-eN-NWR-0019-18-s03.zip › Github/Fig_1/A/populations_pict_B&W.pptx]

## Slide 1
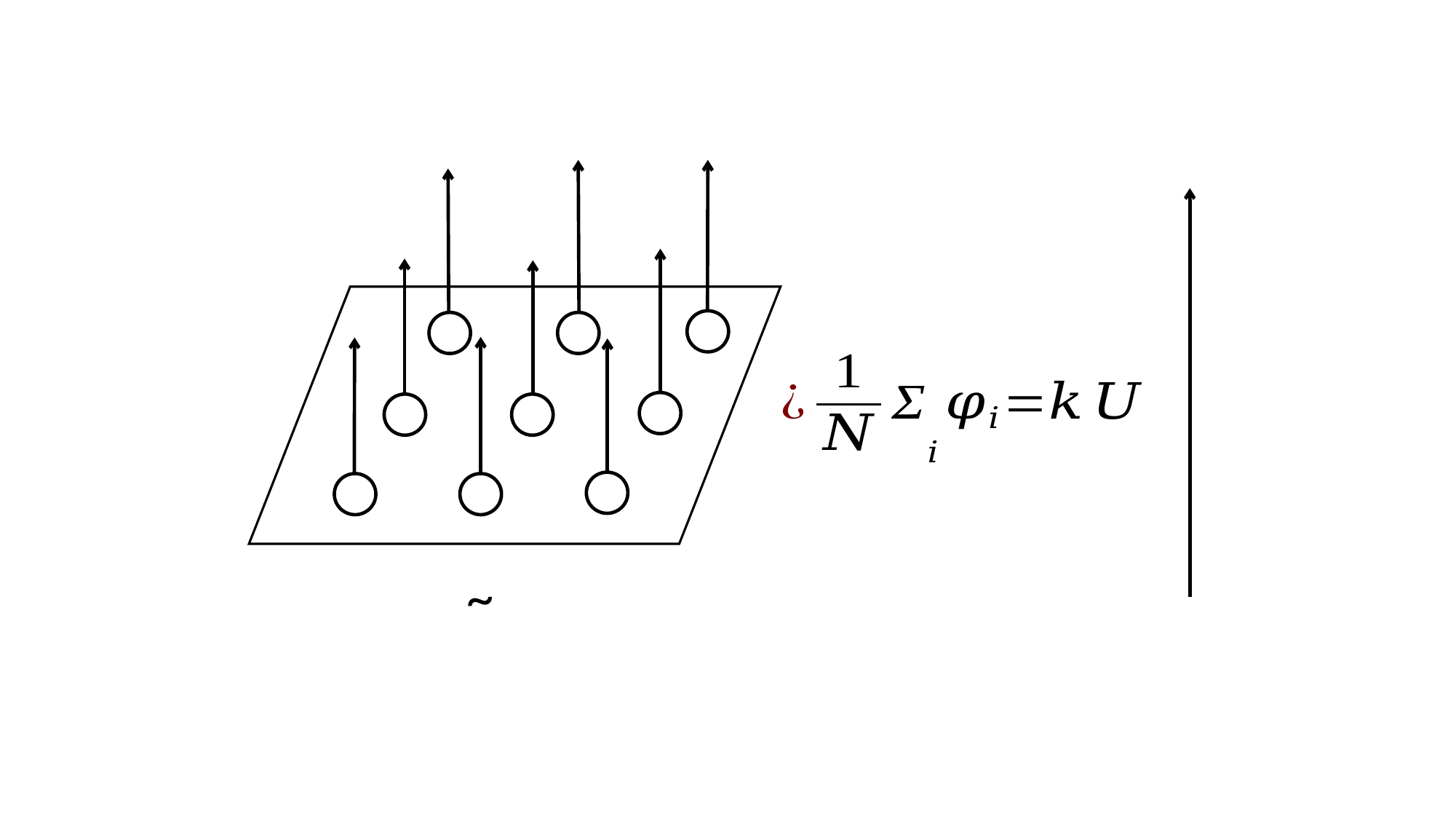

Supplement: Extended Data 1 — The code is available as Extended Data. Download Extended Data, ZIP file [file sup_enu-eN-NWR-0019-18-s03.zip › Github/Fig_1/C/LFP_appr.pptx]
